# Supplementary material for: Efficient accumulation of new irregular monoterpene malonyl glucosides in Nicotiana benthamiana achieved by co-expression of isoprenyl diphosphate synthases and substrate-producing enzymes
Source: Front Plant Sci. 2025 Oct 6;16:1678814. doi: 10.3389/fpls.2025.1678814 (PMC12536018; doi:10.3389/fpls.2025.1678814)
Supplement: Supplementary file 1 [file DataSheet1.pdf]

Supporting Information

Table of contents

S1 Experimental section 4

S1.1 Chemicals and equipment 4

S1.2 Coding sequences 4

Table S1.1 Combinations of the GoldenBraid vectors used in experiments on the biosynthesis of irregular monoterpene malonyl glucosides in *N. benthamiana*. 6

Table S1.2 ^{1}H and ^{13}C NMR data of 1-6 in DMSO-d_6. Values are in ppm. The multiplicities and coupling constants (*J* in Hz) are in parentheses. 10

S2 Results 13

Figure S2.1 GFP (left picture) and mCherry (centre picture) fluorescence in protoplasts of *N. benthamiana*. 13

Figure S2.2 COSY (bold lines) and key HMBC (arrows) correlations (A), and NOESY correlations (B) of 1 in DMSO-d_6. 15

Figure S2.3 ^{1}H NMR spectrum of 1 in DMSO-d_6. The signals representing residual sample impurities are crossed out. 16

Figure S2.4 ^{13}C NMR spectrum of 1 in DMSO-d_6. The signals representing residual sample impurities are crossed out. 17

Figure S2.5 ^{1}H -^{13}C HSQC spectrum of 1 in DMSO-d_6. The signals representing residual sample impurities are crossed out. 18

Figure S2.6 ^{1}H -^{1}H CLIP-COSY spectrum of 1 in DMSO-d_6. 19

Figure S2.7 ^{1}H -^{13}C HMBC spectrum of 1 in DMSO-d_6. 20

Figure S2.8 ^{1}H -^{1}H TOCSY spectrum of 1 in DMSO-d_6. 21

Figure S2.9 ^{1}H -^{1}H NOESY spectrum of 1 in DMSO-d_6. 22

Figure S2.10 COSY (bold lines) and key HMBC (arrows) correlations (A), and NOESY correlations (B) of 2 in DMSO-d_6. 23

Figure S2.11 ^{1}H NMR spectrum of 2 in DMSO-d_6. The signals representing residual sample impurities are crossed out. 24

Figure S2.12 ^{13}C NMR spectrum of 2 in DMSO-d_6. The signals representing residual sample impurities are crossed out. 25

Figure S2.13 ^{1}H -^{13}C HSQC spectrum of 2 in DMSO-d_6. The signals representing residual sample impurities are crossed out. 26

Figure S2.14 ^{1}H -^{1}H CLIP-COSY spectrum of 2 in DMSO-d_6. 27

Figure S2.15 ^{1}H -^{13}C HMBC spectrum of 2 in DMSO-d_6. 28

Figure S2.16 ^{1}H -^{1}H TOCSY spectrum of 2 in DMSO-d_6. 29

Figure S2.17 ^{1}H -^{1}H NOESY spectrum of 2 in DMSO-d_6. 30

Figure S2.18 COSY (bold lines) and key HMBC (arrows) correlations (A), and NOESY correlations (B) of 3 in DMSO-d_6. 31

Figure S2.19 ^{1}H NMR spectrum of 3 in DMSO-d_6. The signals representing residual sample impurities are crossed out. 32

Figure S2.20 ^{13}C NMR spectrum of 3 in DMSO-d_6. The signals representing residual sample impurities are crossed out. 33

Figure S2.21 ^{1}H -^{13}C HSQC spectrum of 3 in DMSO-d_6. The signals representing residual sample impurities are crossed out. 34

Figure S2.22 ^{1}H -^{1}H CLIP-COSY spectrum of 3 in DMSO-d_6. 35

Figure S2.23 ^{1}H -^{13}C HMBC spectrum of 3 in DMSO-d_6. 36

Figure S2.24 ^{1}H -^{1}H TOCSY spectrum of 3 in DMSO-d_6. 37

Figure S2.25 1H -1H NOESY spectrum of 3 in DMSO-d6. 38

Figure S2.26 COSY (bold lines) and key HMBC (arrows) correlations (A), and NOESY correlations (B) of 4 in DMSO-d_6. 39

Figure S2.27 ^{1}H NMR spectrum of 4 in DMSO-d_6. The signals representing residual sample impurities are crossed out. 40

Figure S2.28 ^{13}C NMR spectrum of 4 in DMSO-d_6. The signals representing residual sample impurities are crossed out. 41

Figure S2.29 ^{1}H -^{13}C HSQC spectrum of 4 in DMSO-d_6. The signals representing residual sample impurities are crossed out. 42

Figure S2.30 ^{1}H -^{1}H CLIP-COSY spectrum of 4 in DMSO-d_6. 43

Figure S2.31 ^{1}H -^{13}C HMBC spectrum of 4 in DMSO-d_6. 44

Figure S2.32 ^{1}H -^{1}H TOCSY spectrum of 4 in DMSO-d_6. 45

Figure S2.33 ^{1}H -^{1}H NOESY spectrum of 4 in DMSO-d_6. 46

Figure S2.34 COSY (bold lines) and key HMBC (arrows) correlations (A), and NOESY correlations (B) of 5 in DMSO-d_6. 47

Figure S2.35 ^{1}H NMR spectrum of 5 in DMSO-d_6. The signals representing residual sample impurities are crossed out. 48

Figure S2.36 ^{13}C NMR spectrum of 5 in DMSO-d_6. The signals representing residual sample impurities are crossed out. 49

Figure S2.37 ^{1}H -^{13}C HSQC spectrum of 5 in DMSO-d_6. The signals representing residual sample impurities are crossed out. 50

Figure S2.38 ^{1}H -^{1}H CLIP-COSY spectrum of 5 in DMSO-d_6. 51

Figure S2.39 ^{1}H -^{13}C HMBC spectrum of 5 in DMSO-d_6. 52

Figure S2.40 ^{1}H -^{1}H TOCSY spectrum of 5 in DMSO-d_6. 53

Figure S2.41 ^{1}H -^{1}H NOESY spectrum of 5 in DMSO-d_6........ 54

Figure S2.42 COSY (bold lines) and key HMBC (arrows) correlations (A), and NOESY correlations (B) of 6 in DMSO-d_6. 55

Figure S2.43 ^{1}H NMR spectrum of 6 in DMSO-d_6. The signals representing residual sample impurities are crossed out. 56

Figure S2.44 ^{13}C NMR spectrum of 6 in DMSO-d_6. The signals representing residual sample impurities are crossed out. 57

Figure S2.45 ^{1}H -^{13}C HSQC spectrum of 6 in DMSO-d_6. The signals representing residual sample impurities are crossed out. 58

Figure S2.46 ^{1}H -^{1}H CLIP-COSY spectrum of 6 in DMSO-d_6. 59

Figure S2.47 ^{1}H -^{13}C HMBC spectrum of 6 in DMSO-d_6. 60

Figure S2.48 ^{1}H -^{1}H TOCSY spectrum of 6 in DMSO-d_6. 61

Figure S2.49 ^{1}H -^{1}H NOESY spectrum of 6 in DMSO-d_6........ 62

Figure S2.50 Exemplary HPLC separation of the reaction mixture after MPP-derivatization. The following samples are represented: 63

Figure S2.51 *N. benthamiana* plants expressing StCLDS alone and in combination with tHMGR. The infiltrated leaves are indicated by the arrows 64

S1 Experimental section

S1.1 Chemicals and equipment

Commercial laboratory chemicals, reagents and components of nutrition medium were purchased from the companies Merck (Germany), Roth (Germany), AppliChem (Germany) etc. 3-Methyl-1-phenyl-2-pyrazolin-5-on was ordered from Merck (Germany). Solvents for liquid chromatography were obtained from VWR (Germany) and Roth (Germany).

S1.2 Coding sequences

The sequence encoding LDS from *Lavandula x intermedia* was synthesized according to the GenBank entry JX985358.1. The coding sequence of CDS from *Tanacetum cinerariifolium* was designed based on the GenBank entry JX913536.1, with modifications on the N-terminus of the chloroplast transit peptide (GenBank Acc. No. PX138249). The coding sequence of CLDS from *Streptomyces* sp. CL190 was codon-optimized for *Nicotiana*:

gcattcctgcagttacctacagtttctccaataggaagaattaattctaagcttcttattccctctttctcgtcactccgaacc
tgcaccagcactgcaggaaaacgaatcggagagaggccgaagcttgaacgcttctcccgttcgattcctaagtgct
gtttagcaggtgcagaaaccgaaattgacgaggtgacacccaatcacgtcgcaattatcatagacggacacagaa
agtgggcaaagagtagaggggttacagttcaagagggtcatcaaaccggtgttaacaattggaagcatatcatttcc
cgggcttctcaactcggaatcaagcttctcacaatctgggccttatccccgcagaattttaatcgctctaaaatggaagt
tgacttcttgatgaggatttacgaagatttcctacgatccgatgtcaaagaacttgtcaccagccaacaagacattcaat
tttctgcgattggtgacaaatcaagactcccagaatatctacaagacgcaatatcctacgctgaaggactgagccag
gctaacaagggcatgcatttcatactggcggtagcgtacggcggacgtgaagacatcgtggaggcggccagaaa
gatcgcagccaaagtcgaacacggtatcctacgaccagacgacatcgacgaagctacgttcgaacaacatctgat
gaccaacatcacaaaattcccaagcccggatctactgattagggcagccggtgaacagaggctcagcaacttctttc
tatggcagttgcccttcacagaattctactttacgcctaaattgtttccggattttggcgaggcggatcttctcgacgcgctt
gcctcttaccgctgcaggtatagaggcttcggtgaacgaaaaggaattcatgaa

The sequences encoding IDI from *E. coli* and *Bacillus licheniformis* were synthesized according to the GenBank entries AP026104.1 (range 3694692 to 3695234) and CP014781.1 (range 2437718 to 2438764), respectively. The sequences encoding the mature protein IDI1 without the chloroplast targeting peptide and IPK were amplified from the cDNA of *Arabidopsis thaliana* using the primers based on the GenBank sequences NM_121649.6 for IDI1

Fw: 5'-ACTACGTCTCACTCGAGCCGCTTTCTCAGCCGTC-3';

Rv: 5'-TCTACGTCTCACTCGCTGCGAGCTTGTGAATGG-3'

and AY150412.1 for IPK

Fw: 5'-ACTACGTCTCACTCGAGCCGAGCTGAATATTTCC-3';

Rv: 5'-TCTACGTCTCACTCGCTGCCTTTGAGAATCTGATG-3').

All coding sequences were adapted for the GoldenBraid cloning system by deleting the start and stop codons and addition of nucleotides AGCC on 5'-end and GCAG on 3'-end.

Table S1.1 Combinations of the GoldenBraid vectors used in experiments on the biosynthesis of irregular monoterpene malonyl glucosides in *N. benthamiana*. P35S - 35S CaMV promoter; HisTnos - nopaline synthase gene terminator from *A. tumefaciens* preceded by a sequence encoding 8 histidine residues; ctp – sequence encoding an artificial chloroplast transit peptide

IDS	Auxiliary genes	Product
omega1<br>P35S::*Tc*CDS::HisTnos<br>P35S::p19::Tnos	alpha2	1, 2
omega1<br>P35S::*Tc*CDS::HisTnos<br>P35S::p19::Tnos	alpha2<br>P35S::*Sl*DXS2::HisTnos	1, 2
omega1<br>P35S::*Tc*CDS::HisTnos<br>P35S::p19::Tnos	alpha1<br>P35S::ctp::*At*IDI1::HisTnos	1, 2
omega1<br>P35S::*Tc*CDS::HisTnos<br>P35S::p19::Tnos	alpha1<br>P35S::ctp::*Ec*IDI::HisTnos	1, 2
omega1<br>P35S::*Tc*CDS::HisTnos<br>P35S::p19::Tnos	alpha1<br>P35S::ctp::*Bl*IDI::HisTnos	1, 2
omega1<br>P35S::*Tc*CDS::HisTnos<br>P35S::p19::Tnos	alpha1<br>P35S::ctp::*At*IPK::HisTnos	1, 2
omega1<br>P35S::*Tc*CDS::HisTnos<br>P35S::p19::Tnos	alpha2<br>P35S::tHMGR::HisTnos	1, 2
omega1<br>P35S::*Tc*CDS::HisTnos<br>P35S::p19::Tnos	omega2<br>P35S::ctp::*At*IDI1::HisTnos<br>P35S::*Sl*DXS2::HisTnos	1, 2

omega1<br>P35S::*Tc*CDS::HisTnos<br>P35S::p19::Tnos	omega2<br>P35S::ctp::*Ec*IDI::HisTnos<br>P35S::*Sl*DXS2::HisTnos	1, 2
omega1<br>P35S::*Tc*CDS::HisTnos<br>P35S::p19::Tnos	omega2<br>P35S::ctp::*Bl*IDI::HisTnos<br>P35S::*Sl*DXS2::HisTnos	1, 2
omega1<br>P35S::*Tc*CDS::HisTnos<br>P35S::p19::Tnos	omega2<br>P35S::ctp::*At*IPK::HisTnos<br>P35S::*Sl*DXS2::HisTnos	1, 2
omega1<br>P35S::*Tc*CDS::HisTnos<br>P35S::p19::Tnos	omega2<br>P35S::*At*IDI1::HisTnos<br>P35S::tHMGR::HisTnos	1, 2
omega1<br>P35S::*Tc*CDS::HisTnos<br>P35S::p19::Tnos	omega2<br>P35S::ctp::*Ec*IDI::HisTnos<br>P35S::*Sl*DXS2::HisTnos<br>alpha1<br>P35S::ctp::*At*IPK::HisTnos	1, 2
omega1<br>P35S::*Li*LDS::HisTnos<br>P35S::p19::Tnos	alpha2	3,4
omega1<br>P35S::*Li*LDS::HisTnos<br>P35S::p19::Tnos	alpha2<br>P35S::*Sl*DXS2::HisTnos	3,4
omega1<br>P35S::*Li*LDS::HisTnos<br>P35S::p19::Tnos	alpha1<br>P35S::ctp::*At*IDI1::HisTnos	3,4
omega1<br>P35S::*Li*LDS::HisTnos<br>P35S::p19::Tnos	omega2<br>P35S::ctp::*At*IDI1::HisTnos<br>P35S::*Sl*DXS2::HisTnos	3,4
omega1<br>P35S::ctp::*St*CLDS::HisTnos	alpha2	5,6

P35S::p19::Tnos		
omega1<br>P35S::ctp::*St*CLDS::HisTnos<br>P35S::p19::Tnos	alpha2<br>P35S::*Sl*DXS2::HisTnos	5,6
omega1<br>P35S::ctp::*St*CLDS::HisTnos<br>P35S::p19::Tnos	alpha1<br>P35S::ctp::*At*IDI1::HisTnos	5,6
omega1<br>P35S::ctp::*St*CLDS::HisTnos<br>P35S::p19::Tnos	omega2<br>P35S::ctp::*At*IDI1::HisTnos<br>P35S::*Sl*DXS2::HisTnos	5,6
omega1<br>P35S::*St*CLDS::HisTnos<br>P35S::p19::Tnos	alpha2	5,6
omega1<br>P35S::*St*CLDS::HisTnos<br>P35S::p19::Tnos	alpha2<br>P35S::tHMGR::HisTnos	5,6
omega1<br>P35S::*St*CLDS::HisTnos<br>P35S::p19::Tnos	alpha1<br>P35S::*At*IDI1::HisTnos	5,6
omega1<br>P35S::*St*CLDS::HisTnos<br>P35S::p19::Tnos	alpha1<br>P35S::*At*IPK::HisTnos	5,6
omega1<br>P35S::*St*CLDS::HisTnos<br>P35S::p19::Tnos	omega2<br>P35S::*At*IDI1::HisTnos<br>P35S::tHMGR::HisTnos	5,6
omega1<br>P35S::*St*CLDS::HisTnos<br>P35S::p19::Tnos	omega2<br>P35S::*Ec*IDI::HisTnos<br>P35S::tHMGR::HisTnos	5,6
omega1<br>P35S::*St*CLDS::HisTnos<br>P35S::p19::Tnos	omega2<br>P35S::*Bl*IDI::HisTnos<br>P35S::tHMGR::HisTnos	5,6

omega1<br>P35S::*St*CLDS::HisTnos<br>P35S::p19::Tnos	omega2<br>P35S::*At*IPK::HisTnos<br>P35S::tHMGR::HisTnos	5,6
omega1<br>P35S::*St*CLDS::HisTnos<br>P35S::p19::Tnos	alpha2<br>P35S::*Sl*DXS2::HisTnos	5,6
omega1<br>P35S::*St*CLDS::HisTnos<br>P35S::p19::Tnos	omega2<br>P35S::ctp::*At*IDI1::HisTnos<br>P35S::*Sl*DXS2::HisTnos	5,6

Table S1.2 ^{1}H and ^{13}C NMR data of 1-6 in DMSO-d_6. Values are in ppm. The multiplicities and coupling constants (*J* in Hz) are in parentheses.

**Position**	**^{1}H NMR**						**^{13}C NMR**					
	**1**	**2**	**3**	**4**	**5**	**6**	**1**	**2**	**3**	**4**	**5**	**6**
1	3.82 (dd, 1H, *J* = 11, 8.7 Hz); 3.43 (dd, 1H, *J* = 11, 5.7 Hz)	3.79 (t, *J* = 10.7, 8.1 Hz, 1H); 3.51 – 3.47 (m, 1H)	3.44 (dd, 1H, *J* = 9.8, 6.6 Hz); 3.63 (dd, 1H, *J* = 9.8, 7.1 Hz)	3.44 (dd, 1H, *J* = 9.4, 6.2 Hz); 3.66 (dd, 1H, *J* = 9.4, 7.4 Hz)	4.23 (d, *J* = 11.4 Hz, 1H); 3.97 (d, *J* = 11.4 Hz, 1H)	4.13 – 4.08 (m, 2H)	68.56	68.83	70.80	70.74	66.73	67.35
2	0.75 (dt, *J* = 8.5, 5.6 Hz, 1H)	0.81 – 0.77 (m, 1H)	2.30 (m, 1H)	2.29 (m, 1H)	-	-	31.8	31.64	46.45	46.54	124.58	124.99
3	1.10 – 1.07 (m, 1H)	1.11 – 1.08 (m, 1H)	1.98 (m, 1H); 2.2 (dt, *J* = 13.5, 6.4 Hz, 1H)	1.99 (m, 1H); 2.2 (dt, *J* = 13.7, 6.4 Hz, 1H)	2.14 (d, *J* = 17.4 Hz, 1H); 1.95 (d, *J* = 17.4 Hz, 1H)	2.14 (d, *J* = 17.2 Hz, 1H); 2.01 (d, *J* = 17.2 Hz, 1H)	27.56	27.62	28.21	28.14	24.95	24.68
4	4.86 (d, *J* = 8.2 Hz, 1H)	4.86 (d, *J* = 8.2 Hz, 1H)	5.02 (t, 1H, *J* = 7.0 Hz)	5.02 (t, 1H, *J* = 7.1 Hz)	1.28 (t, *J* = 6.6 Hz, 2H)	1.27 (t, *J* = 6.5 Hz, 2H)	123.91	123.94	122.48	122.57	35.12	35.16
5	-	-	-	-	-	-	131.71	131.49	131.36	131.22	28.77	28.74
6	1.62 (s, 3H)	1.63 (s, 3H)	1.63 (s, 3H)	1.63 (s, 3H)	1.73 (s, 2H)	1.71 (s, 2H)	18.12	18.14	25.63	25.62	45.58	45.58
7	1.65 (s, 3H)	1.65 (s, 3H)	1.56 (s, 3H)	1.56 (s, 3H)	-	-	25.43	25.42	17.77	17.78	130.76	129.85
8	-	-	-	-	1.62 (s, 3H)	1.60 (s, 3H)	21.79	21.53	145.49	145.58	18.82	18.87
9	1.07 (s, 3H)	1.07 (s, 3H)	4.73 (s, 1H); 4.69 (s, 1H)	4.73 (s, 1H); 4.68 (s, 1H)	0.86 (s, 3H)	0.85 (s, 3H)	21.22	21.14	111.81	111.68	28.08	28.02
10	0.99 (s,	0.98 (s,	1.64 (s,	1.64 (s,	0.86 (s,	0.85 (s,	22.37	22.36	20.12	20.19	28.12	28.25

	3H)	3H)	3H)	3H)	3H)	3H)						
Glc-1'	4.13 (d, *J* = 7.9 Hz, 1H)	4.31 (d, *J* = 7.7 Hz, 1H)	4.14 (d, *J* = 7.8 Hz, 1H)	4.31 (d, *J* = 7.6 Hz, 1H)	4.03 (d, *J* = 7.9 Hz, 1H)	4.24 (d, *J* = 7.7 Hz, 1H)	102.27	101.02	102.92	101.36	100.43	99.69
2'	2.96 (t, *J* = 9.4, 7.9 Hz, 1H)	3.30 – 3.26 (m, 1H)	2.95 (t, *J* = 9.3, 7.8 Hz, 1H)	3.31 – 3.27 (m, 1H)	2.97 (t, *J* = 9.1, 8.0 Hz, 1H)	3.31 – 3.28 (m, 1H)	73.36	81.68	73.4	81.4	73.22	81.51
3'	3.14 (t, *J* = 9.4, 8.9 Hz, 1H)	3.38 (t, *a* = 9.1 Hz, 1H)	3.14 (t, *J* = 8.9 Hz, 1H)	3.39 (t, *J* = 9.0 Hz, 1H)	3.11 (t, *J* = 9.4, 8.8 Hz, 1H)	3.41 – 3.35 (m, 1H)	76.41	75.51	76.33	75.58	76.47	75.76
4'	3.09 (t, *J* = 9.3, 8.9 Hz, 1H)	3.16 – 3.13 (m, 1H)	3.09 (t, *J* = 10.6, 9.3 Hz, 1H)	3.19 – 3.16 (m, 1H)	3.07 (t, *J* = 9.4, 8.8 Hz, 1H)	3.18 – 3.14 (m, 1H)	70.10	69.72	70.0	69.87	70.20	69.99
5'	3.31 – 3.27 (m, 1H)	3.36 – 3.33 (m, 1H)	3.30 (m, 1H)	3.37 – 3.34 (m, 2H)	3.24 (ddd, *J* = 8.9, 6.9, 1.8 Hz, 1H)	3.34 – 3.31 (m, 1H)	73.69	73.71	73.75	73.53	73.89	73.63
6'	4.16 – 4.05 (m, 2H)	4.14 – 4.10 (m, 1H); 4.07 (d, *J* = 11.6 Hz, 1H)	4.08-4.12 (m, 2H)	4.16 – 4.06 (m, 2H)	4.18 (dd, *J* = 11.8, 1.8 Hz, 1H); 4.06 (dd, *J* = 11.8, 6.9 Hz, 1H)	4.15 (d, *J* = 11.6 Hz, 0H); 4.09 (dd, *J* = 11.6, 6.5 Hz, 1H)	63.51	63.29	63.43	63.33	63.58	63.30
Malonyl C=O_a	-	-	-	-	-	-	169.19	169.72	169.7	169.60	169.31	169.54
CH_2	2.94 – 2.91 (m, 2H)	2.92 – 2.88 (m, 2H)	2.92 – 2.88 (m, 2H)	2.95 – 2.90 (m, 2H)	2.96 – 2.94 (m, 2H)	2.94 – 2.90 (m, 2H)	45.61	45.98	45.86	45.69	45.32	45.63
C=O_b	-	-	-	-	-	-	168.43	167.19	167.63	168.15	168.08	168.13
Glc-1''		4.40 (d, *J* = 7.9 Hz, 1H)		4.38 (d, *J* = 7.7 Hz, 1H)		4.39 (d, *J* = 7.8 Hz, 1H)		103.9		104.11		104.01
2''		2.99 (t, *J* = 8.3 Hz, 2H)		2.98 (t, *J* = 8.4 Hz, 1H)		2.98 (t, *J* = 8.3 Hz, 1H)		74.92		75.10		75.07
3''		3.10 –		3.17 –		3.17 –		76.06		76.01		76.01

	3.16 (m, 1H)	3.10 (m, 1H)	3.11 (m, 1H)			
4''	3.10 – 3.16 (m, 1H)	3.17 – 3.10 (m, 1H)	3.15 – 3.11 (m, 1H)	69.76	69.77	69.72
5''	3.06 – 3.03 (m, 1H)	3.03 – 3.00 (m, 1H)	3.05 – 3.02 (m, 1H)	77.02	77.10	77.01
6''	3.62 (d, *J* = 11.9 Hz, 1H); 3.51 – 3.47 (m, 1H)	3.61 (d, *J* = 11.5 Hz, 1H); 3.50 – 3.48 (m, 1H)	3.60 (d, *J* = 11.6 Hz, 1H); 3.51 – 3.47 (m, 1H)	60.72	60.75	60.8

S2 Results

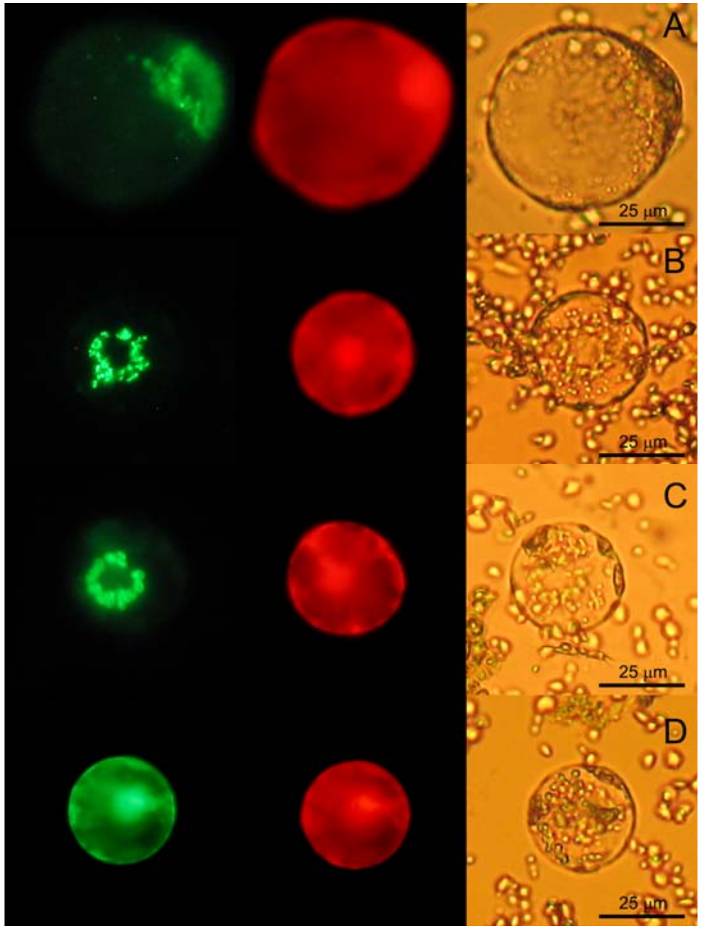

Figure S2.1 GFP (left picture) and mCherry (centre picture) fluorescence in protoplasts of *N. benthamiana* (right picture, visible light), transiently co-expressing fused CDS::GFP gene and mCherry gene (A); fused LDS::GFP gene and mCherry gene (B); ctp::GFP and

mCherry genes (C); GFP and mCherry genes (D). GFP and mCherry fluorescence was monitored by means of the Axioskop 40 microscope (Carl Zeiss), equipped with the Filter Set 38 (excitation BP 470/40, beam splitter FT 495, emission BP 525/50; selective detection of GFP fluorescence) and with the Filter Set 31 (excitation BP 565/30, beam splitter FT 585, emission BP 620/60; selective detection of mCherry fluorescence), and the Canon PC1201 camera. For protoplasts isolation, the explants were cut out of *N. benthamiana* leaves on the 4th day after infiltration with the corresponding *Agrobacterium* strain, shredded and incubated overnight in the mixture of cellulitic enzyme solution, containing 3 % cellulose Onozuca R10 and 1 % macerozyme, and W5 medium, containing 125 mM CaCl2,154 mM NaCl, 5.4 mM KCl, 5.6 mM glucose, in the proportion of 1 to 4, and supplemented with cefotaxime to the final concentration of 500 mg l^{-1}. The samples were incubated overnight at 30 ^{o}C on a shaker at 70 rpm. The protoplast suspension was filtered through Miracloth material and centrifuged at 100 x g for 5 min. The pellet was resuspended in 1 ml of W5 medium.

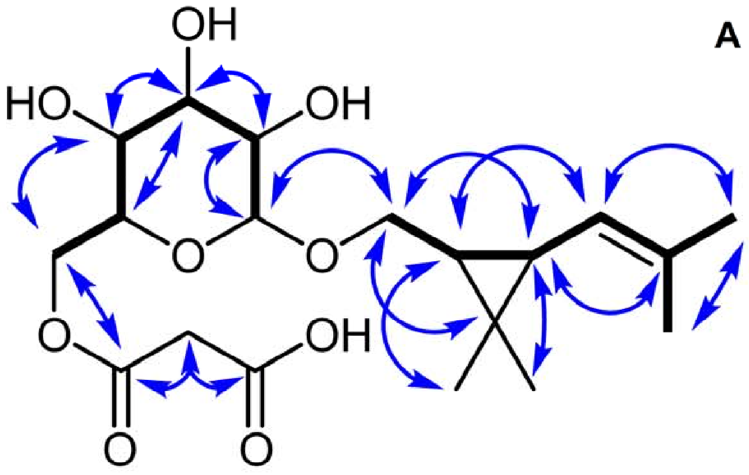

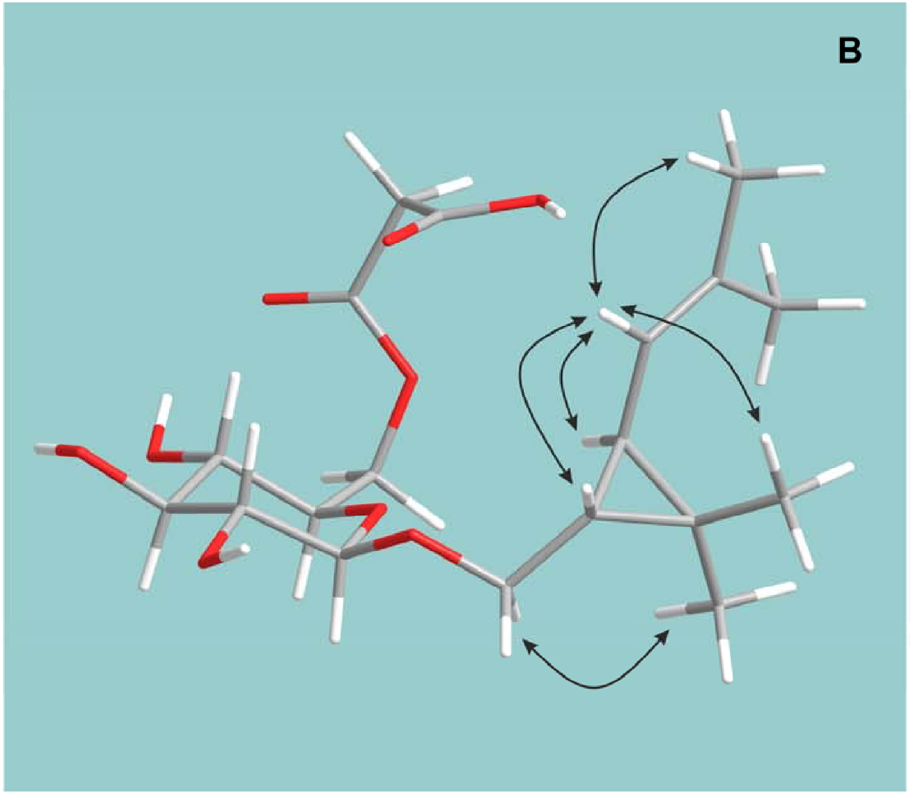

Figure S2.2 COSY (bold lines) and key HMBC (arrows) correlations (A), and NOESY correlations (B) of **1** in DMSO-d_6.

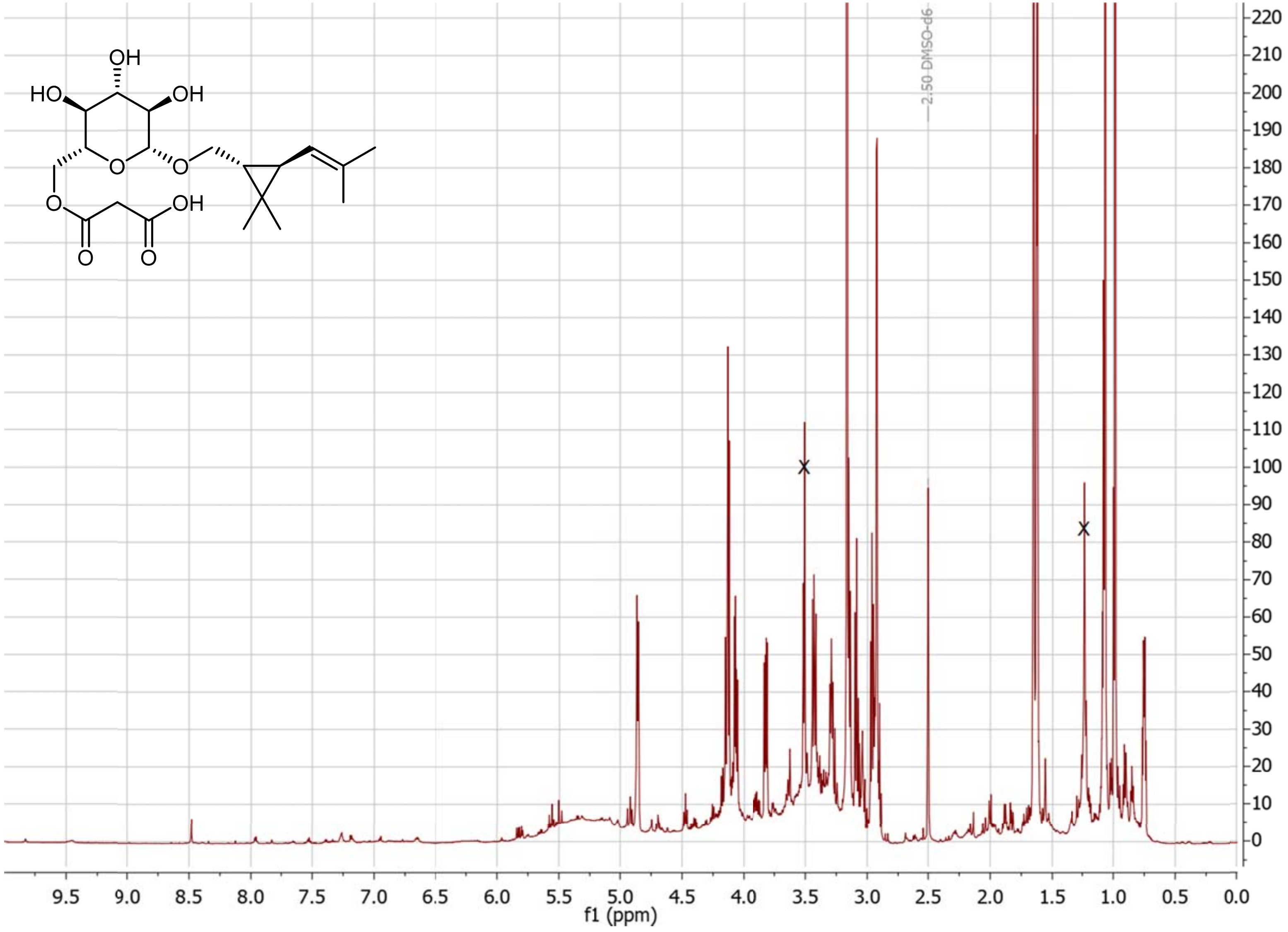

Figure S2.3 ^{1}H NMR spectrum of **1** in DMSO-d_6. The signals representing residual sample impurities are crossed out.

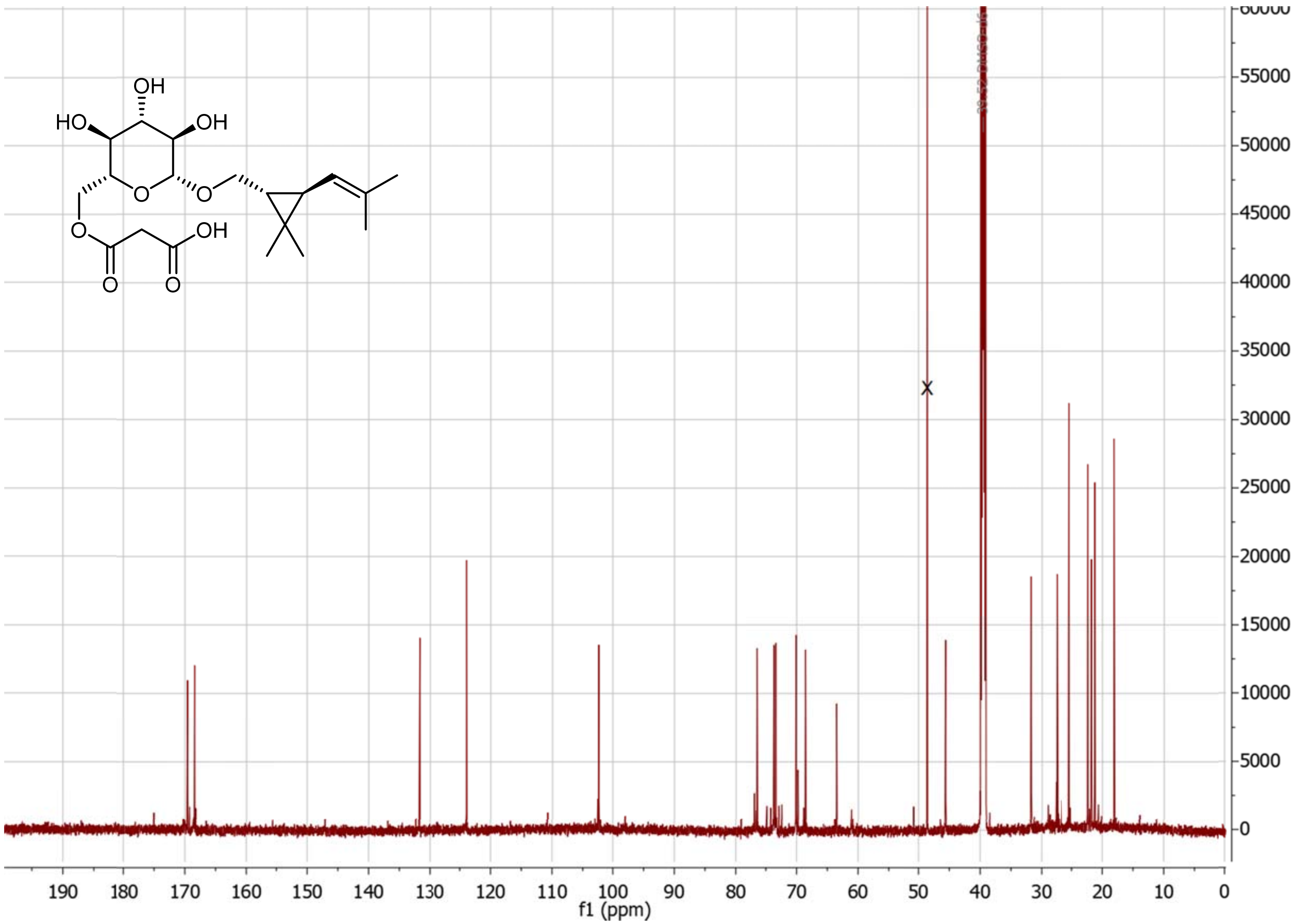

Figure S2.4 ^{13}C NMR spectrum of **1** in DMSO-d_6. The signals representing residual sample impurities are crossed out.

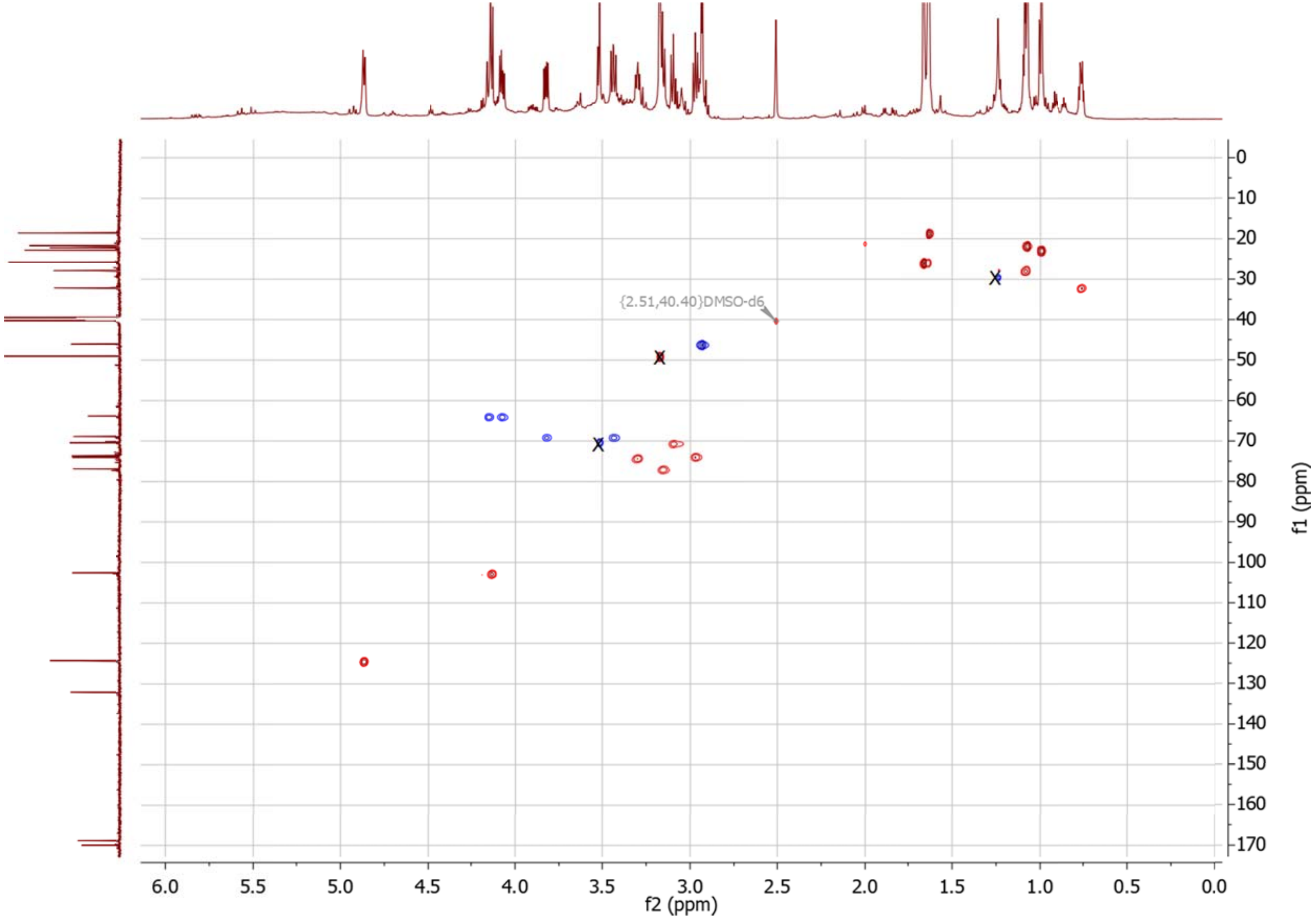

Figure S2.5 ^{1}H -^{13}C HSQC spectrum of **1** in DMSO-d_6. The signals representing residual sample impurities are crossed out.

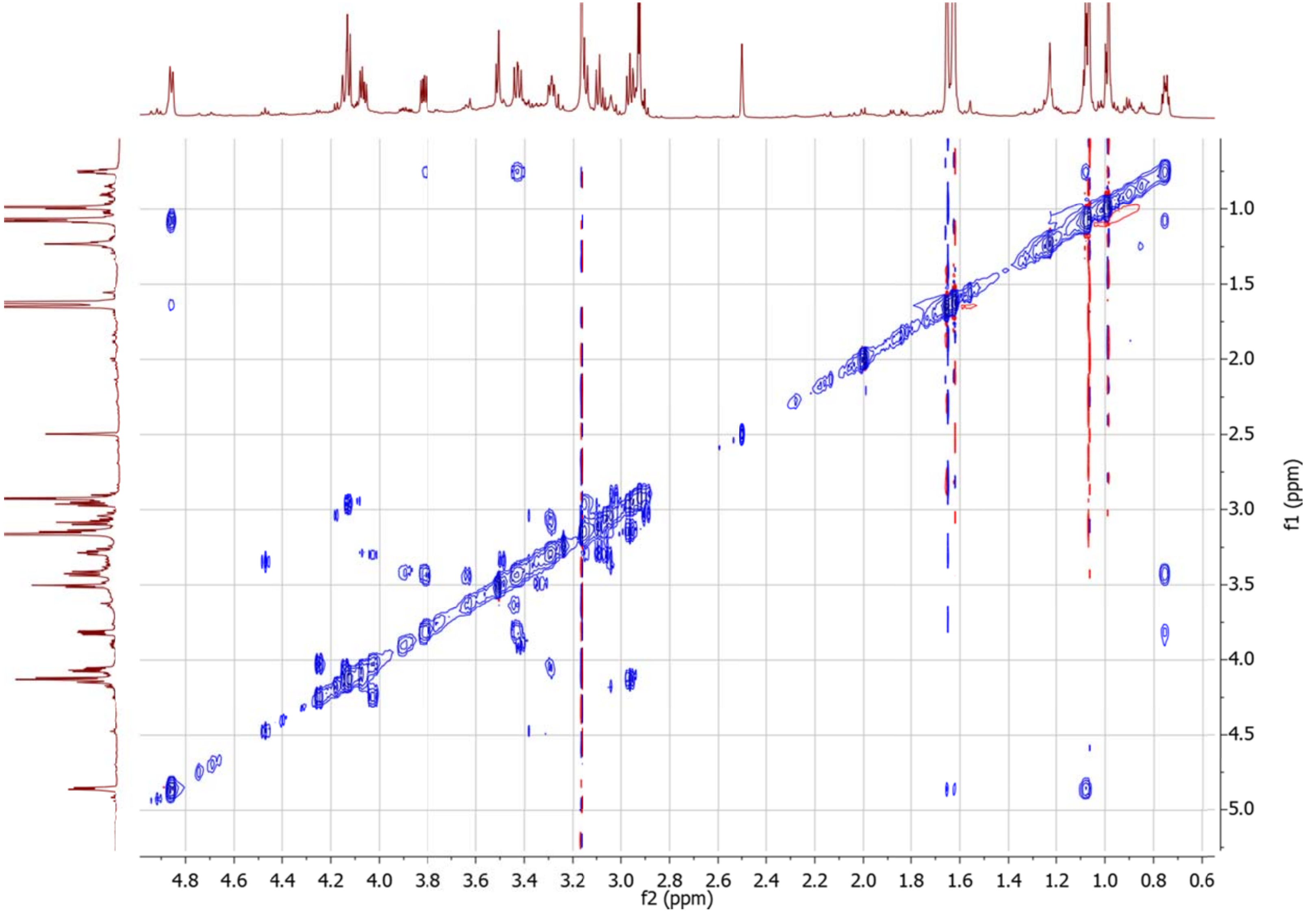

Figure S2.6 ^{1}H -^{1}H CLIP-COSY spectrum of **1** in DMSO-d_6.

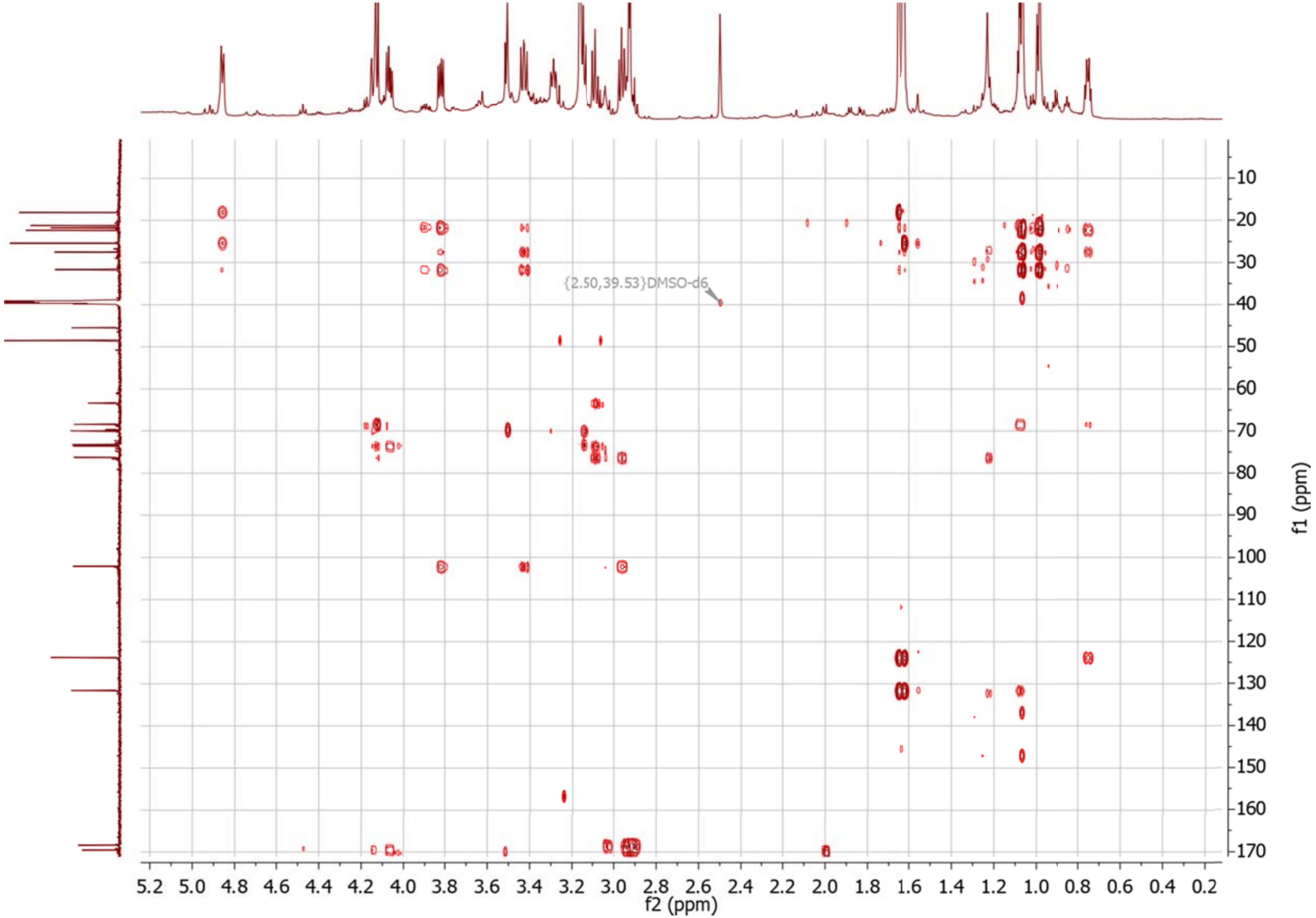

Figure S2.7 ^{1}H -^{13}C HMBC spectrum of **1** in DMSO-d_6.

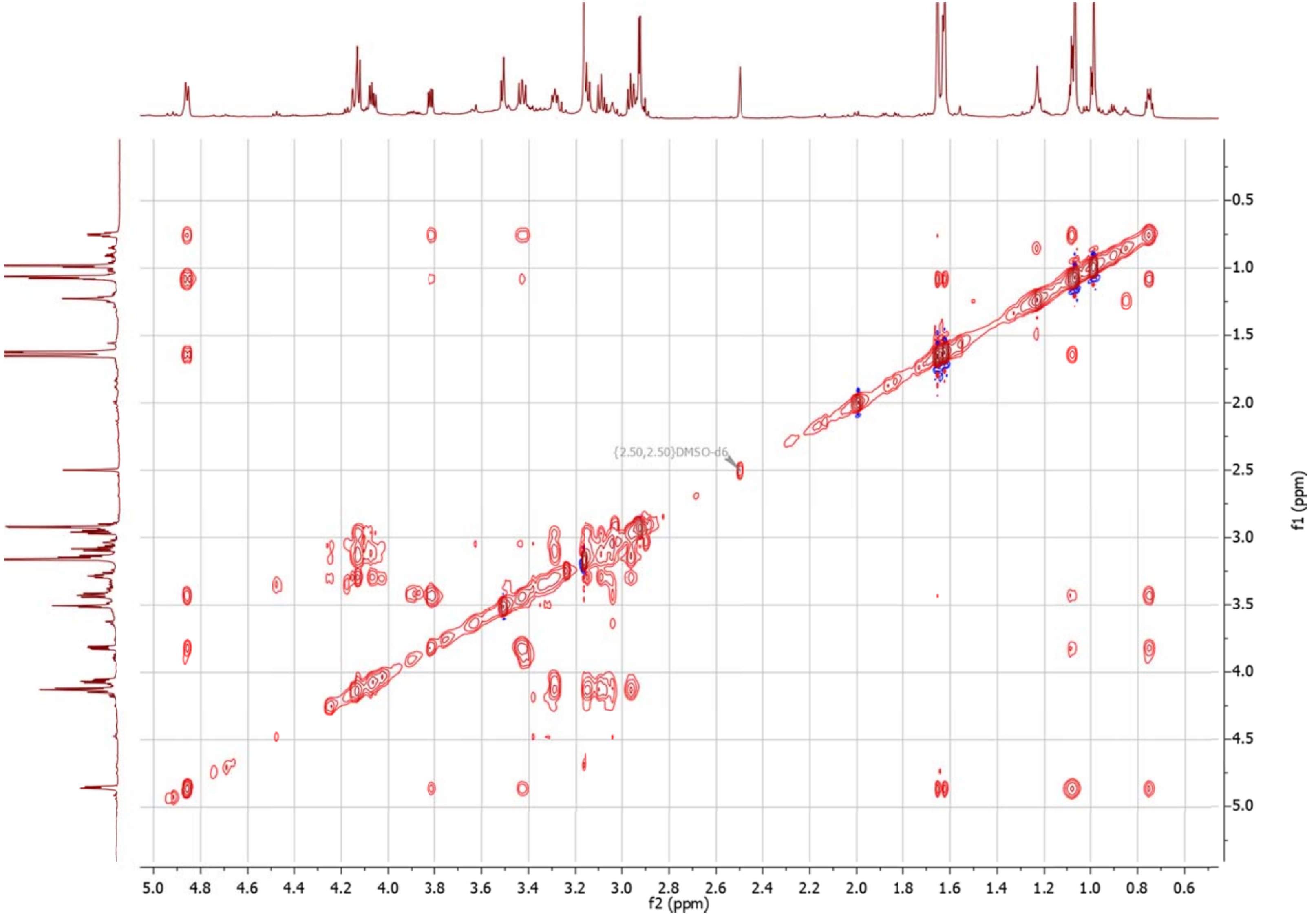

Figure S2.8 ^{1}H -^{1}H TOCSY spectrum of **1** in DMSO-d_6.

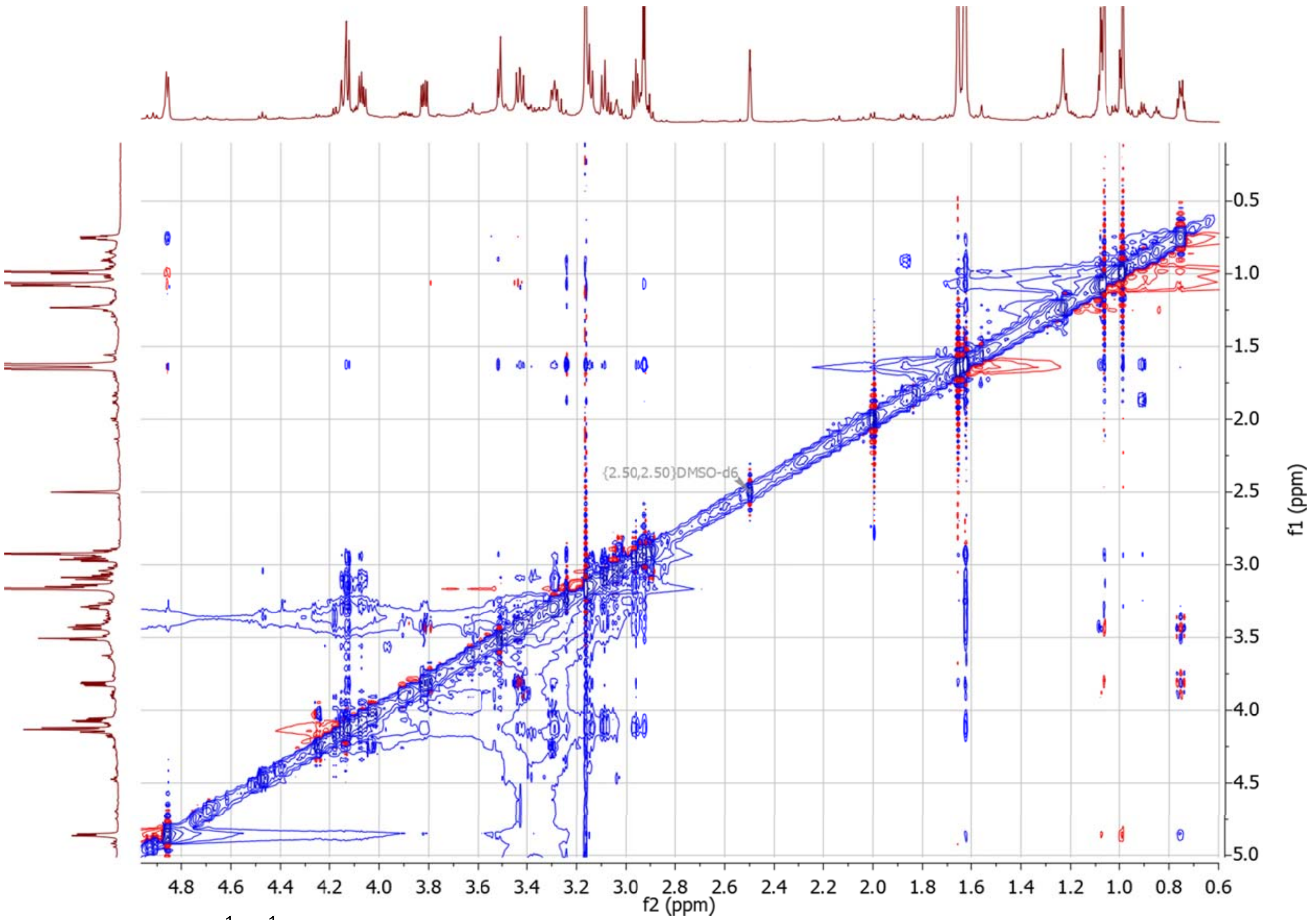

Figure S2.9 ^{1}H -^{1}H NOESY spectrum of **1** in DMSO-d_6.

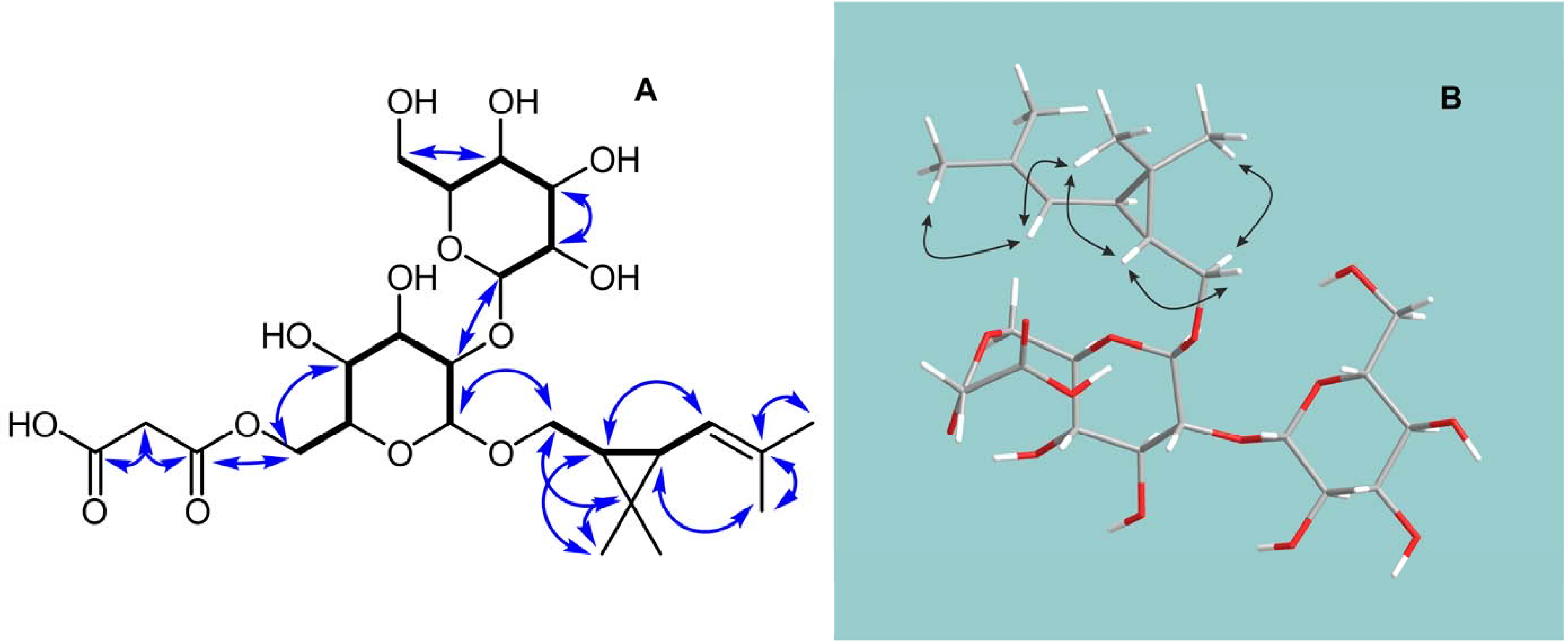

Figure S2.10 COSY (bold lines) and key HMBC (arrows) correlations (A), and NOESY correlations (B) of **2** in DMSO-d_6.

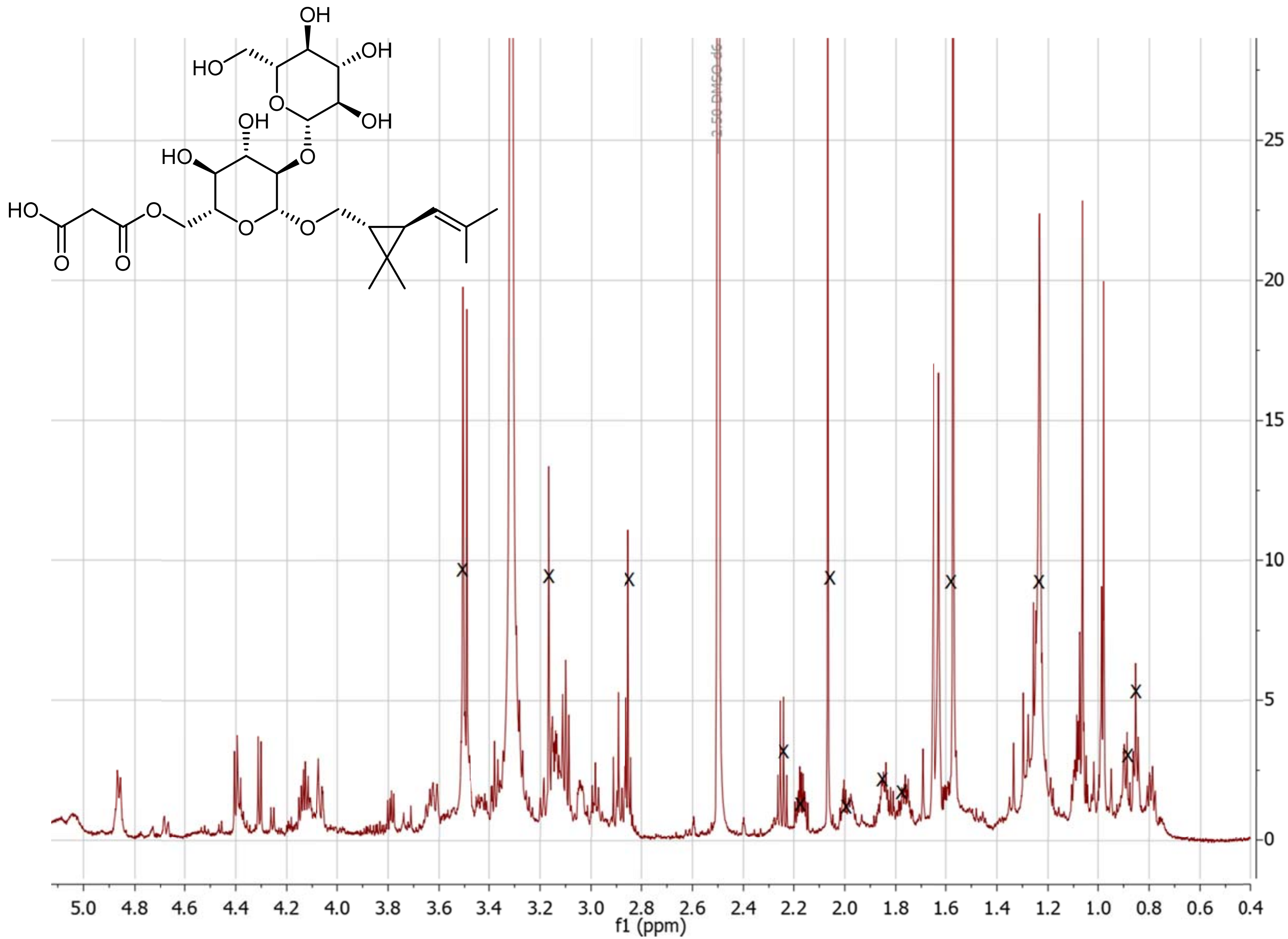

Figure S2.11 ^{1}H NMR spectrum of **2** in DMSO-d_6. The signals representing residual sample impurities are crossed out.

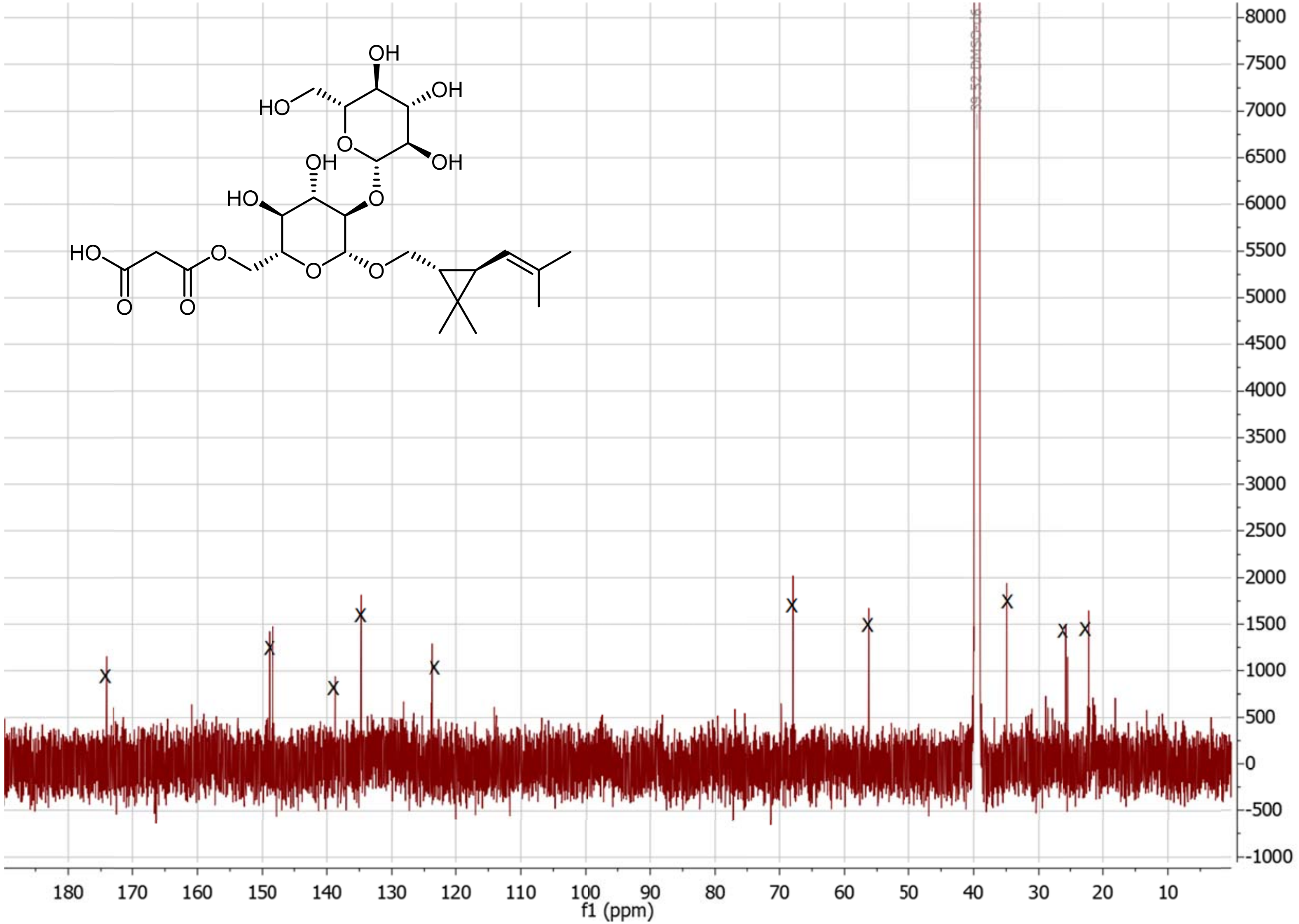

Figure S2.12 ^{13}C NMR spectrum of **2** in DMSO-d_6. The signals representing residual sample impurities are crossed out.

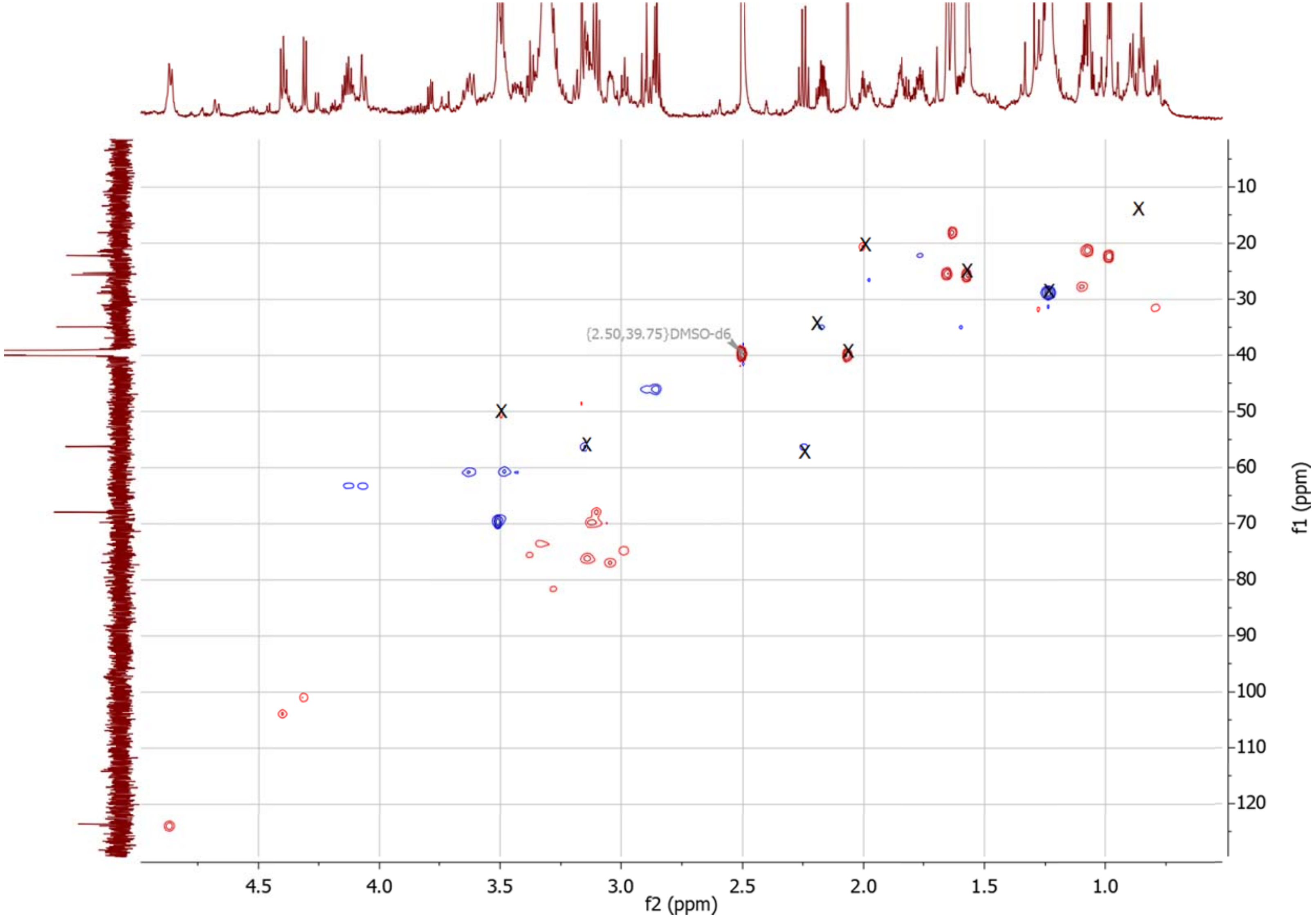

Figure S2.13 ^{1}H -^{13}C HSQC spectrum of **2** in DMSO-d_6. The signals representing residual sample impurities are crossed out.

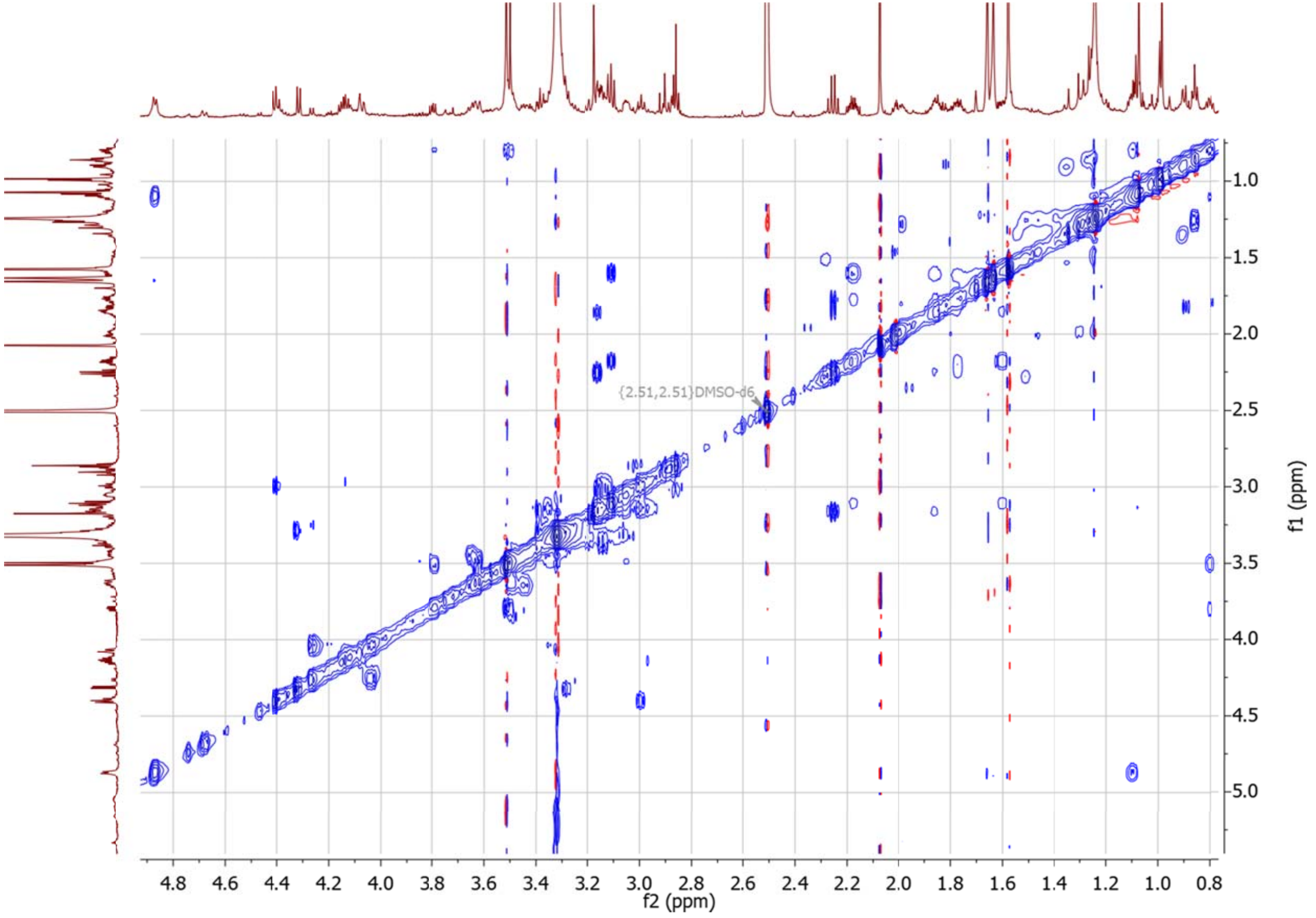

Figure S2.14 ^{1}H -^{1}H CLIP-COSY spectrum of **2** in DMSO-d_6.

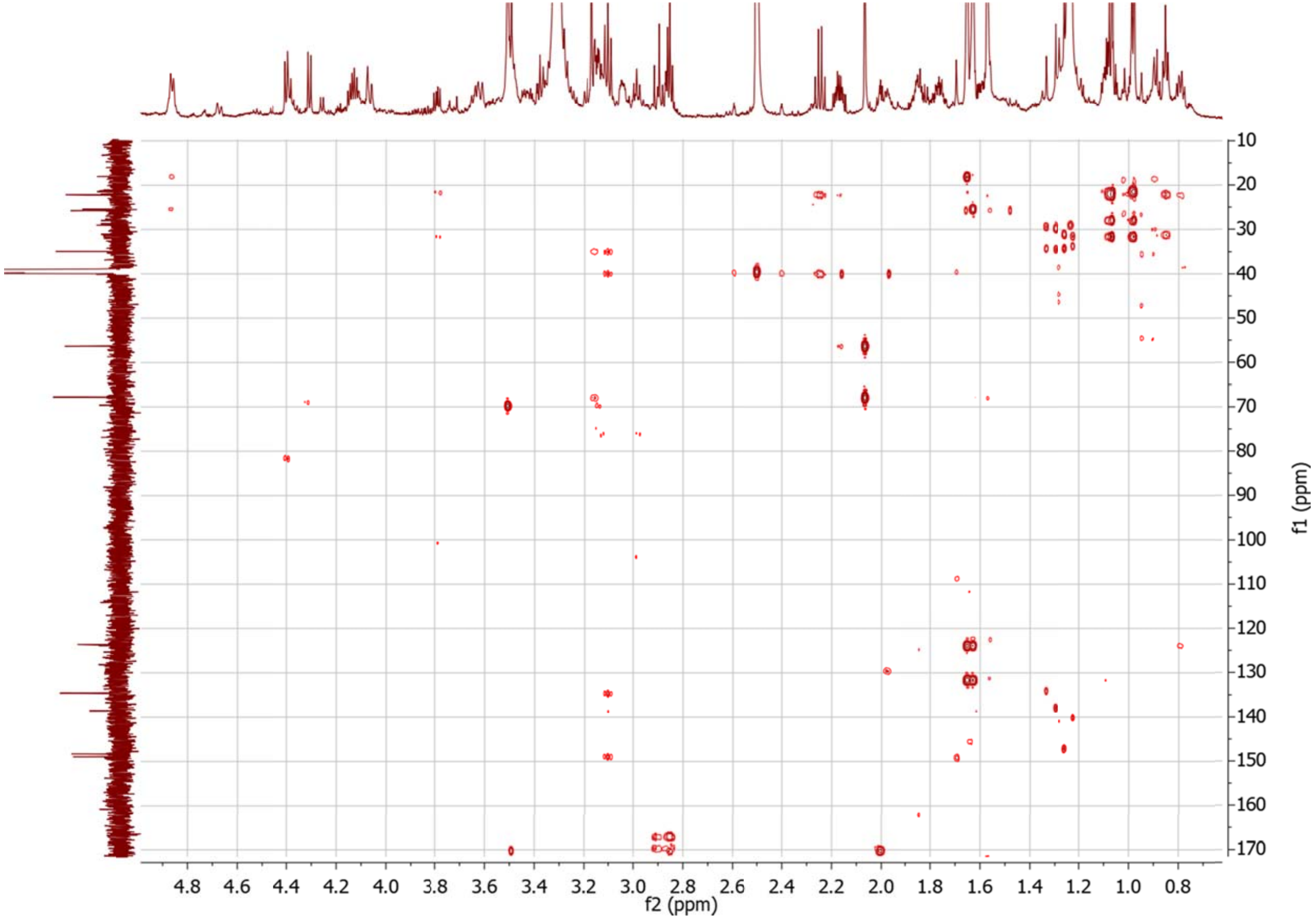

Figure S2.15 ^{1}H -^{13}C HMBC spectrum of **2** in DMSO-d_6.

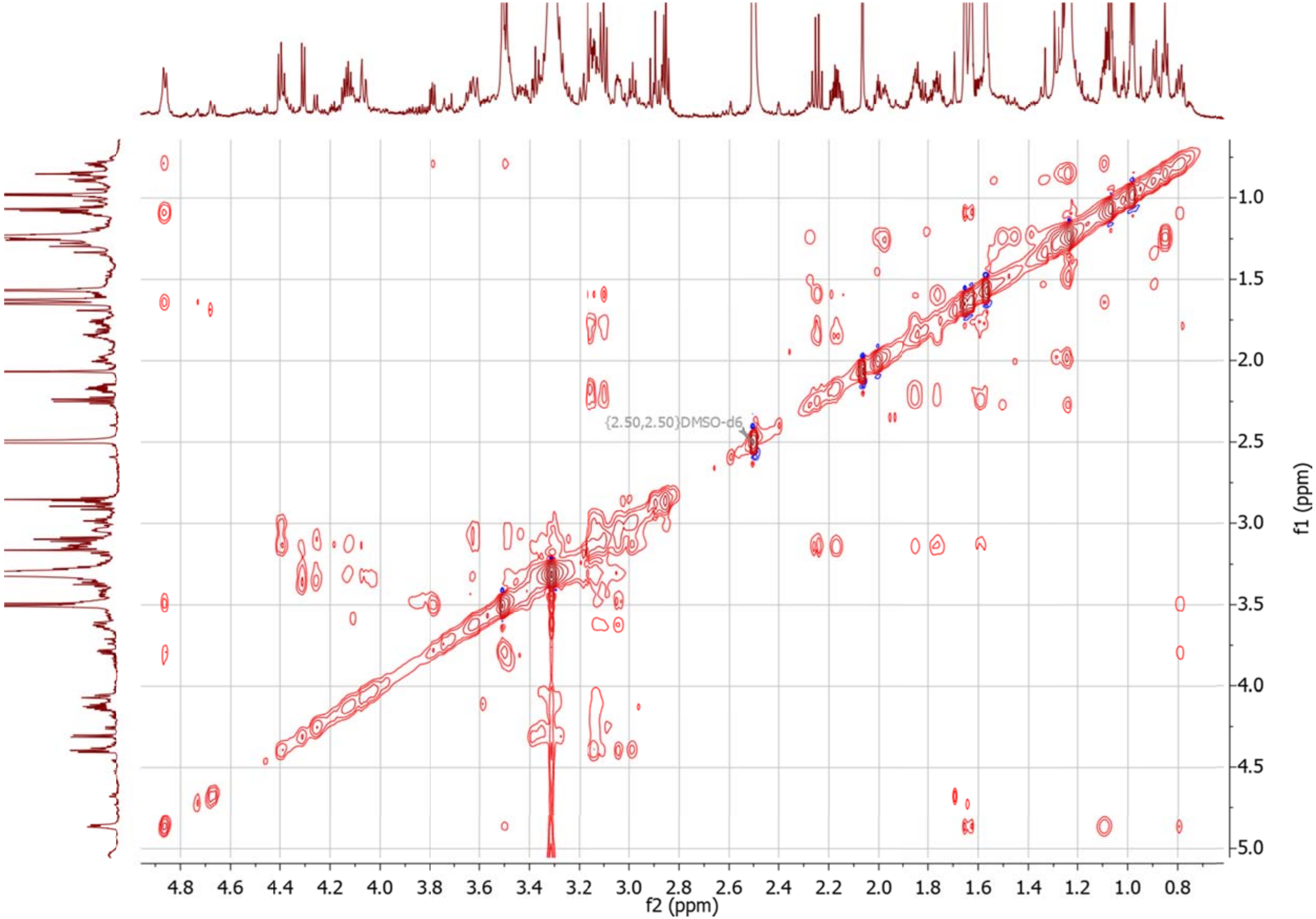

Figure S2.16 ^{1}H -^{1}H TOCSY spectrum of **2** in DMSO-d_6.

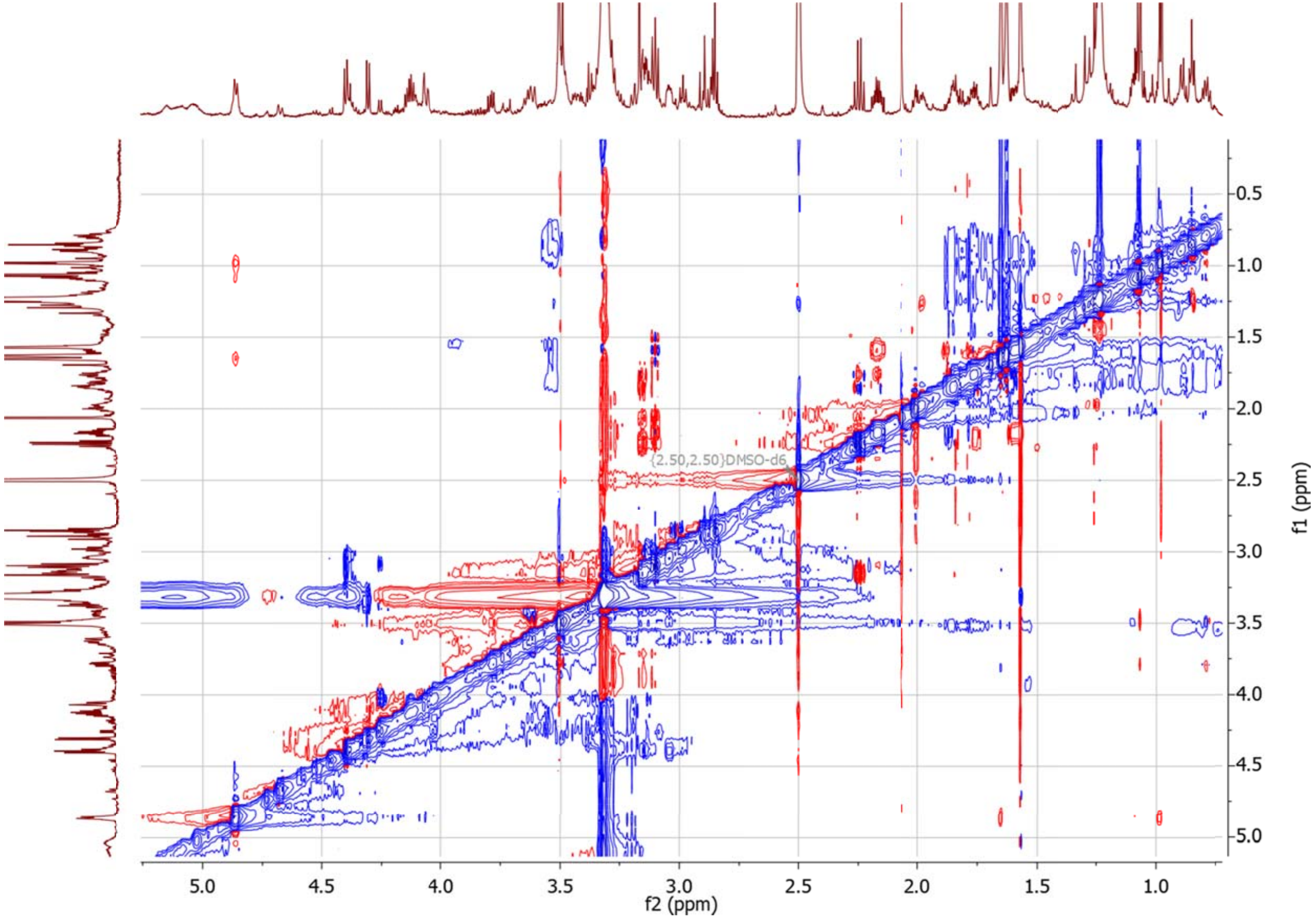

Figure S2.17 ^{1}H -^{1}H NOESY spectrum of **2** in DMSO-d_6.

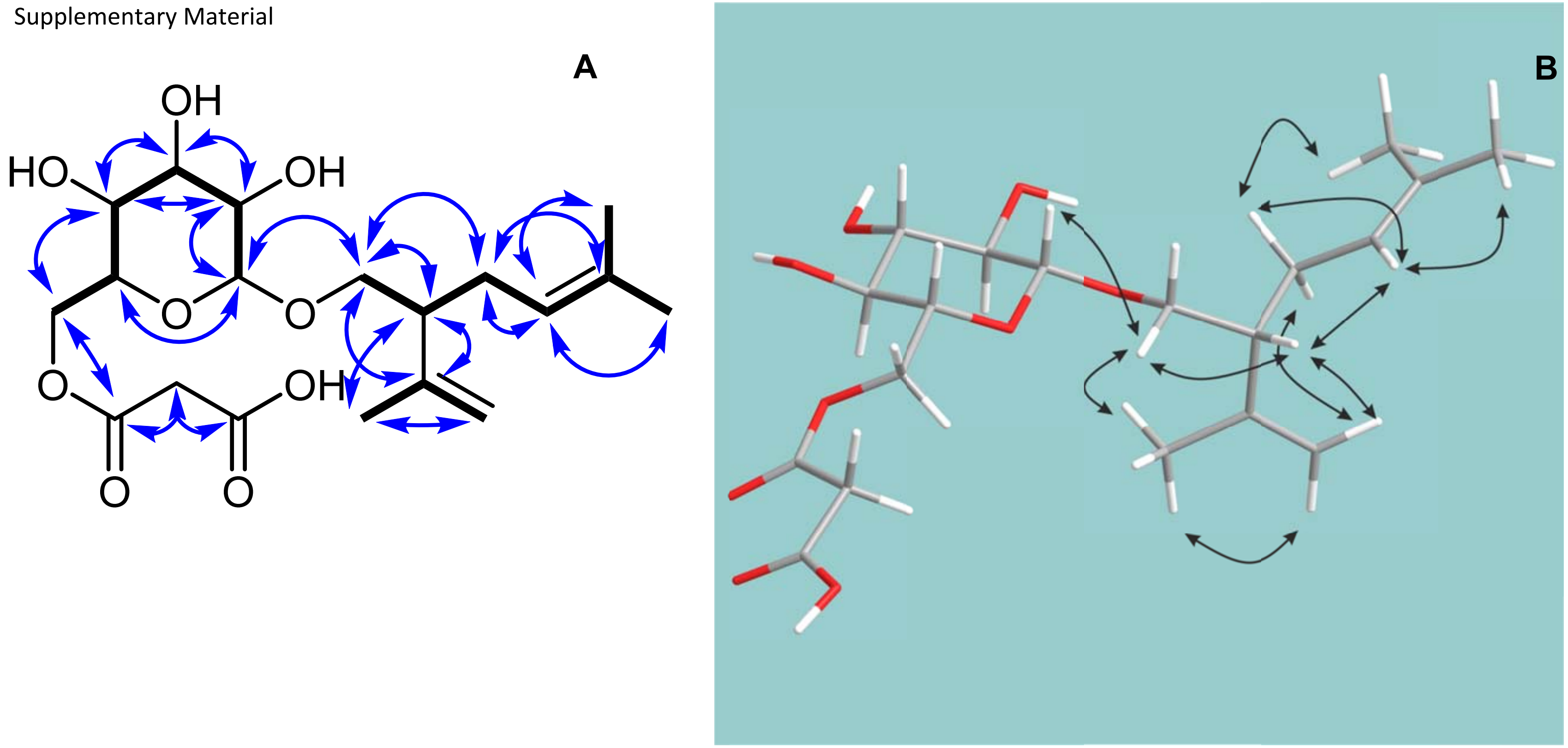

Figure S2.18 COSY (bold lines) and key HMBC (arrows) correlations (A), and NOESY correlations (B) of **3** in DMSO-d_6.

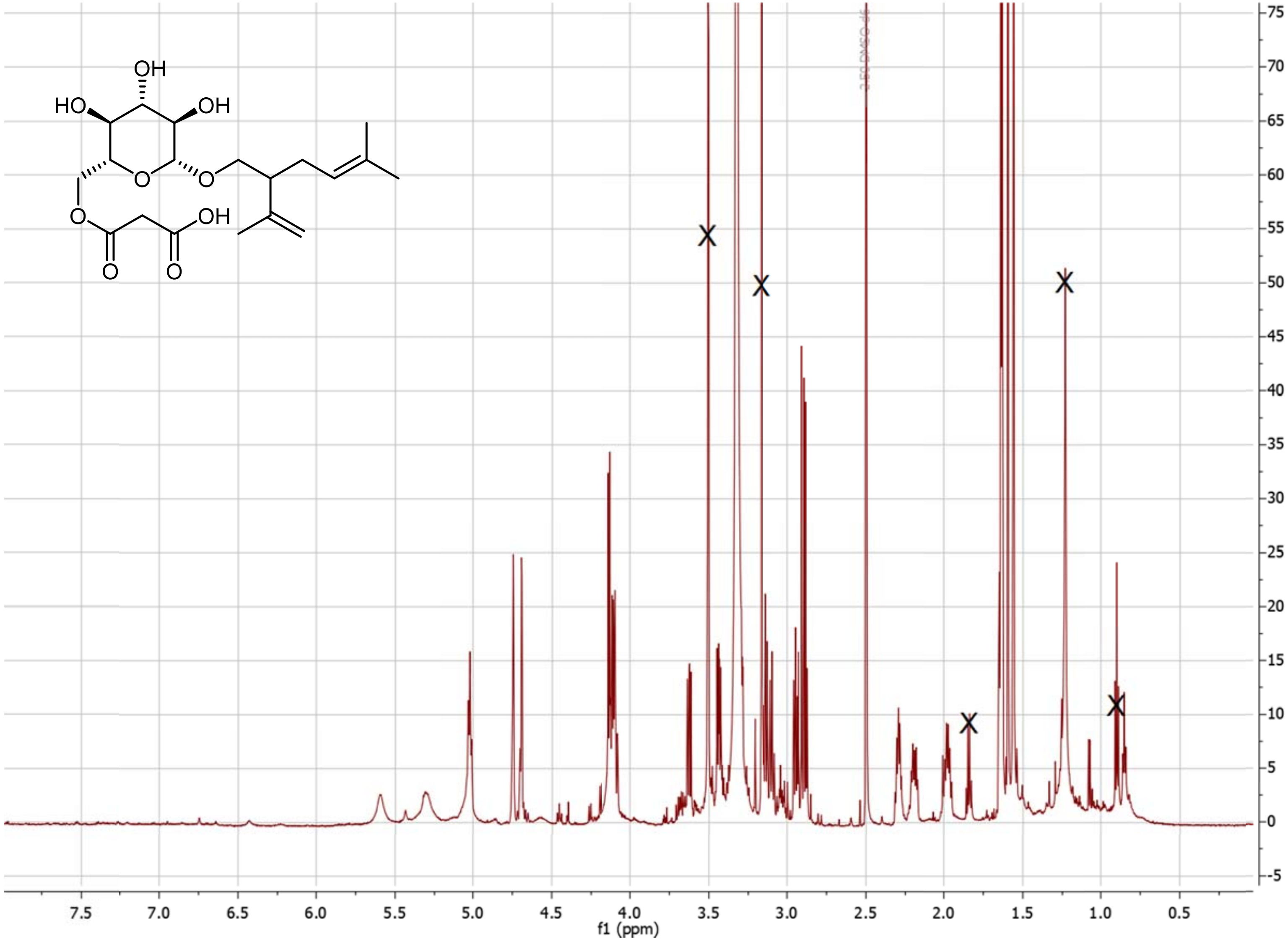

Figure S2.19 ^{1}H NMR spectrum of **3** in DMSO-d_6. The signals representing residual sample impurities are crossed out.

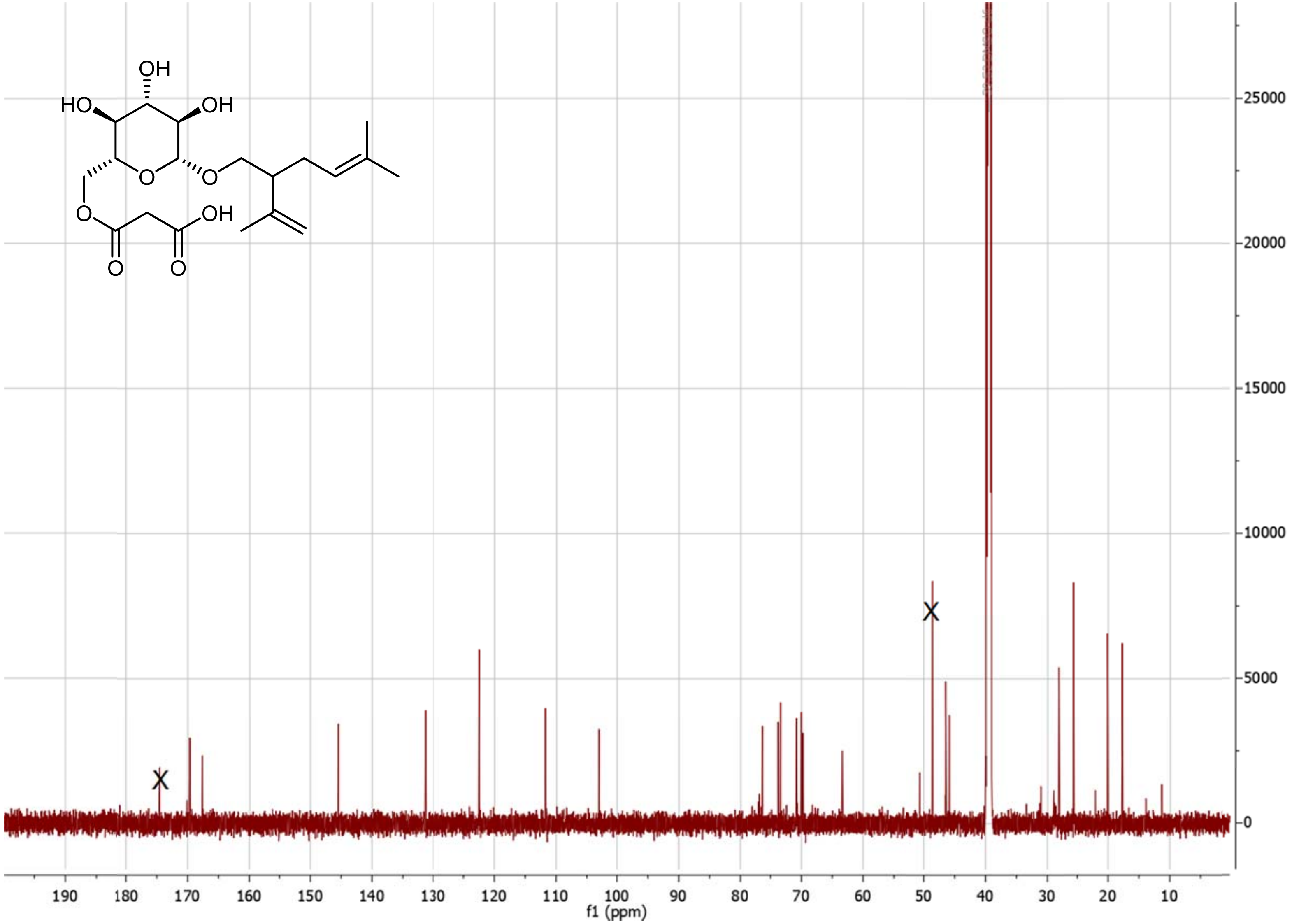

Figure S2.20 ^{13}C NMR spectrum of **3** in DMSO-d_6. The signals representing residual sample impurities are crossed out.

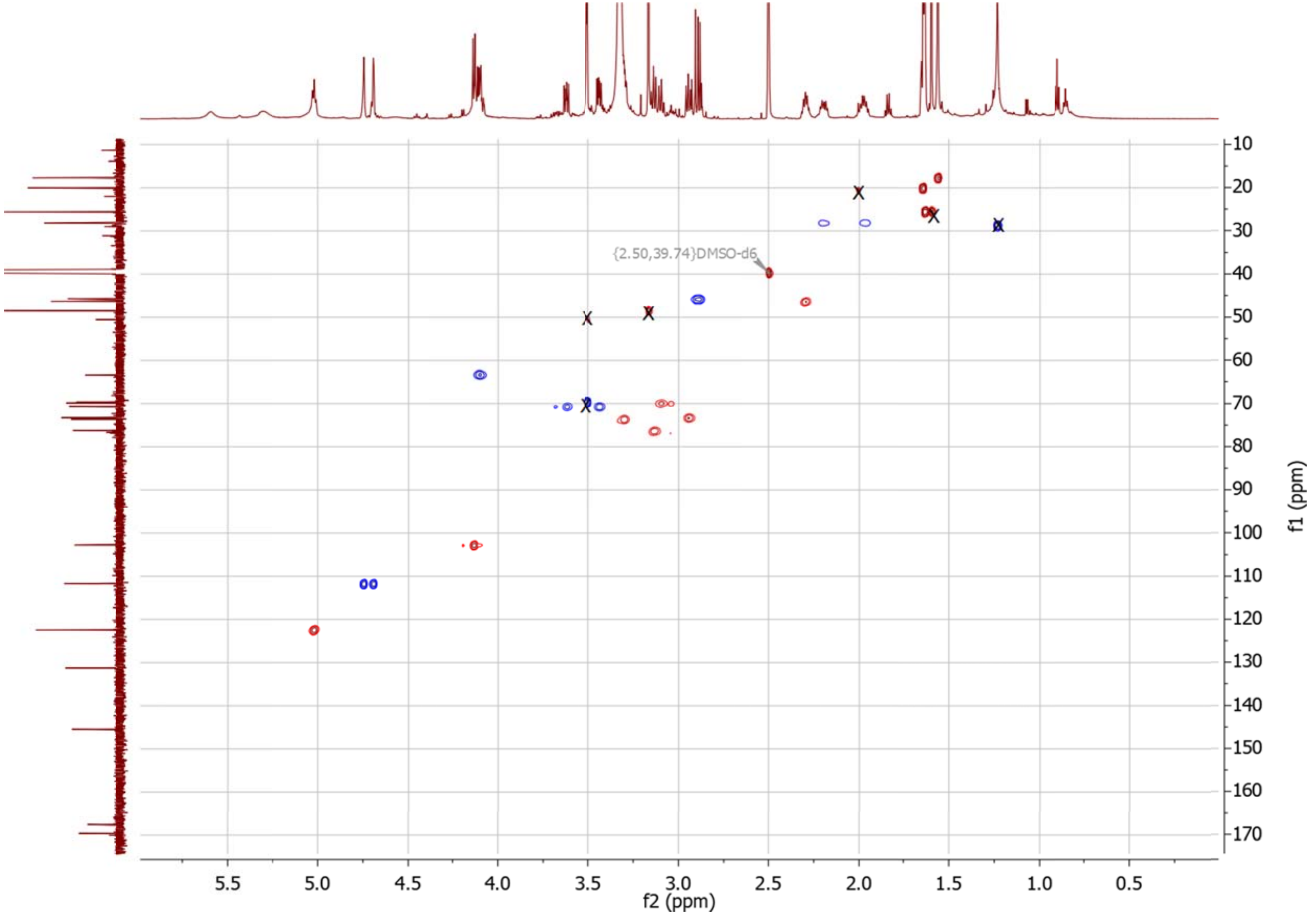

Figure S2.21 ^{1}H -^{13}C HSQC spectrum of **3** in DMSO-d_6. The signals representing residual sample impurities are crossed out.

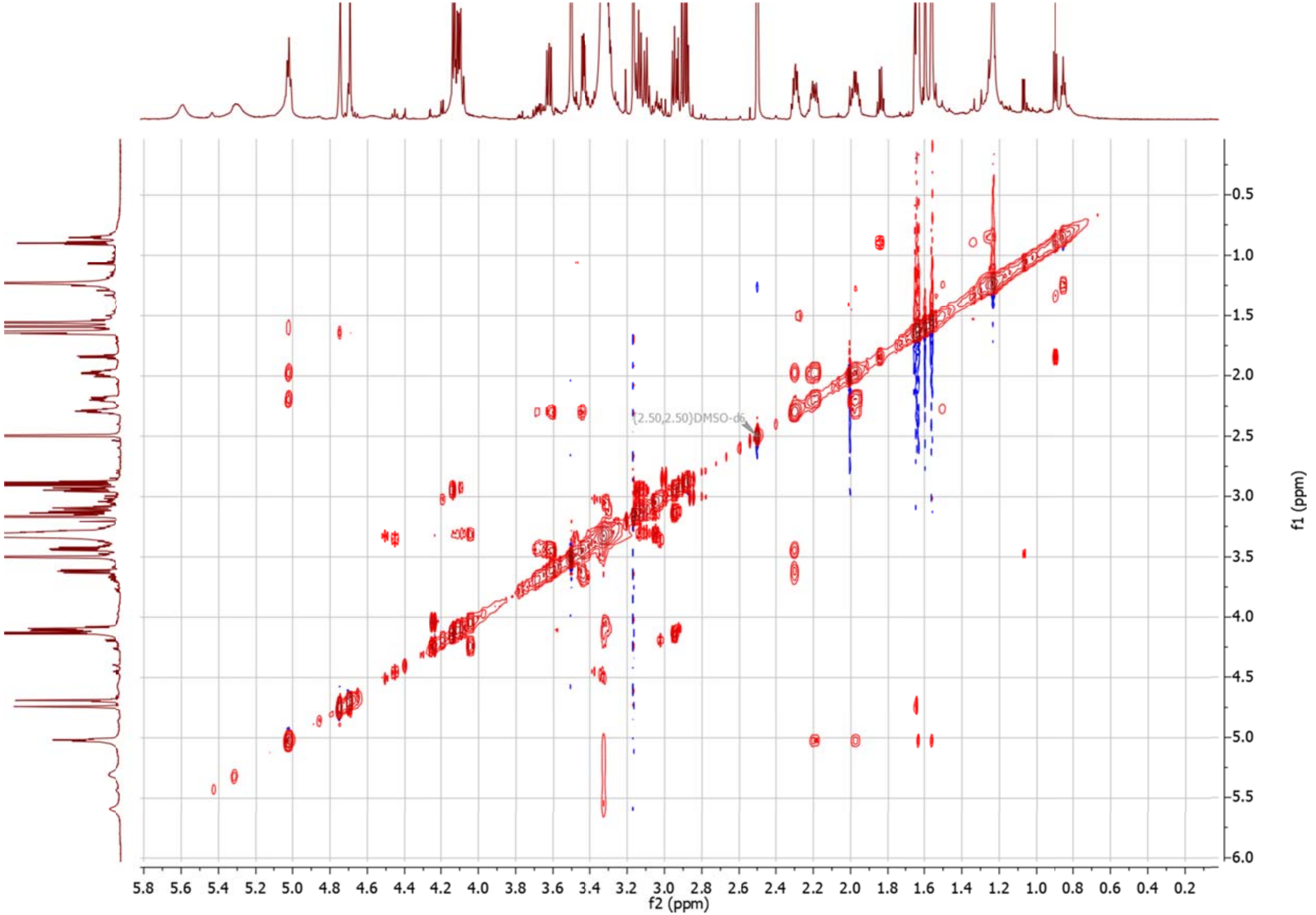

Figure S2.22 ^{1}H -^{1}H CLIP-COSY spectrum of **3** in DMSO-d_6.

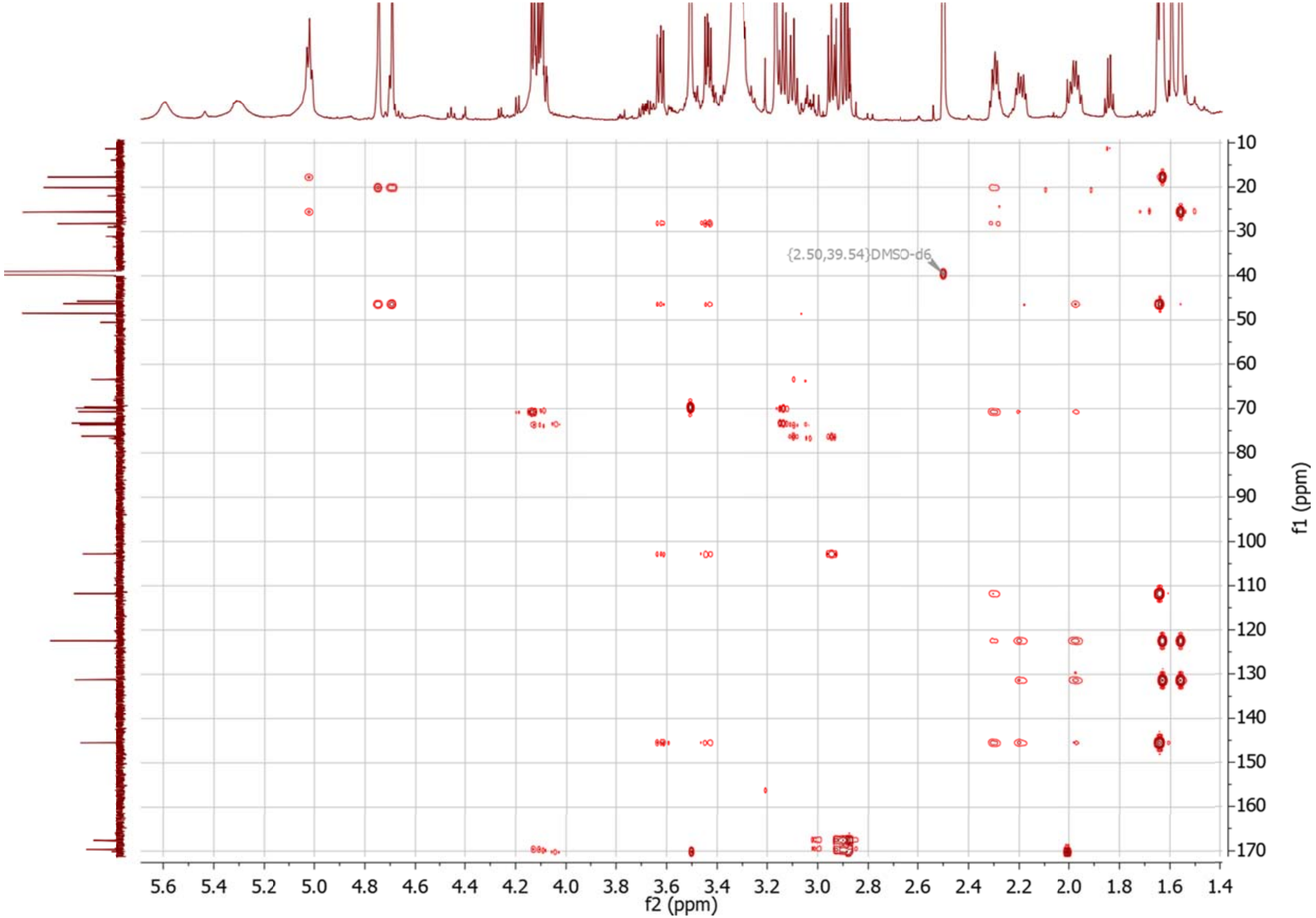

Figure S2.23 ^{1}H -^{13}C HMBC spectrum of **3** in DMSO-d_6.

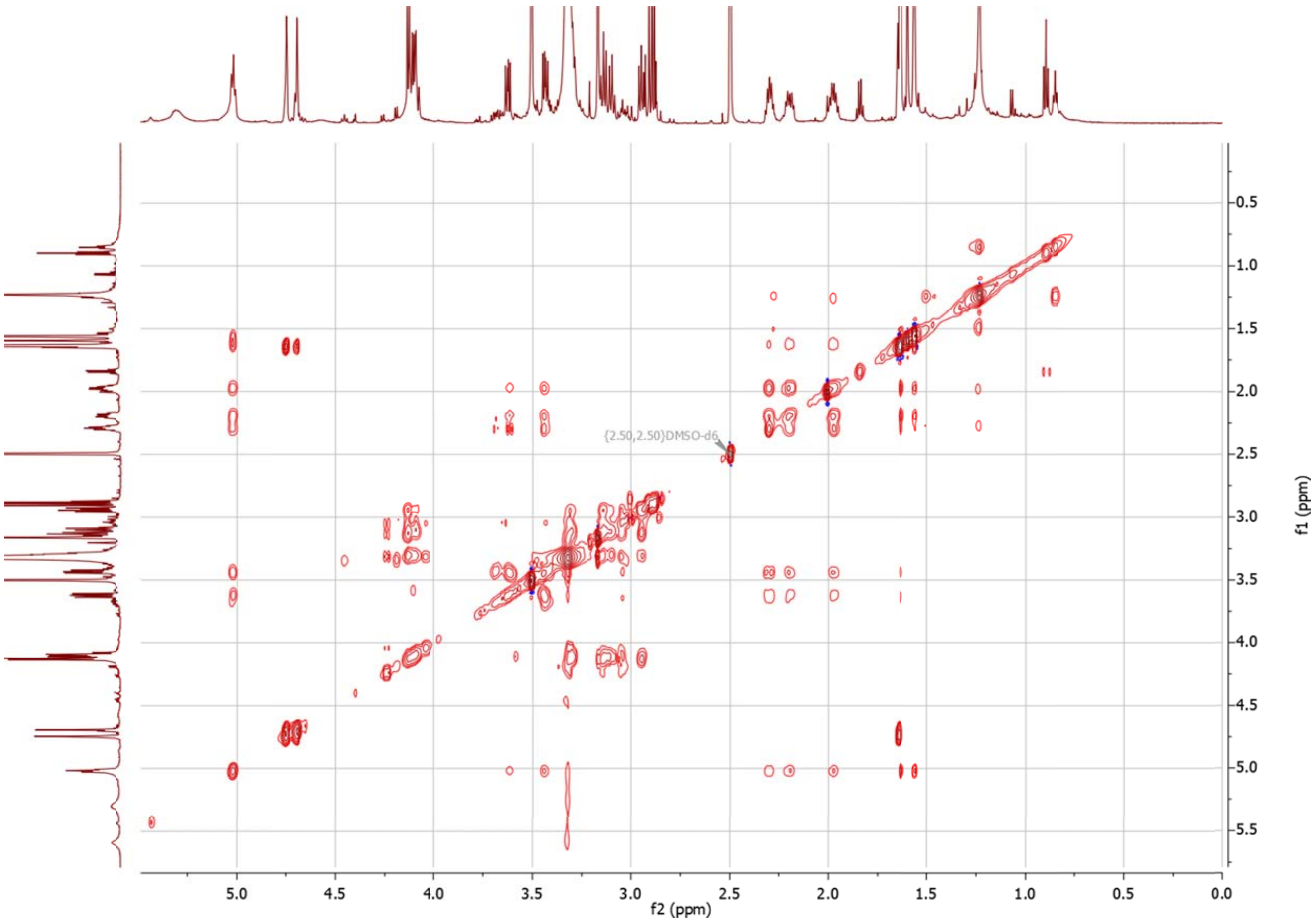

Figure S2.24 ^{1}H -^{1}H TOCSY spectrum of **3** in DMSO-d_6.

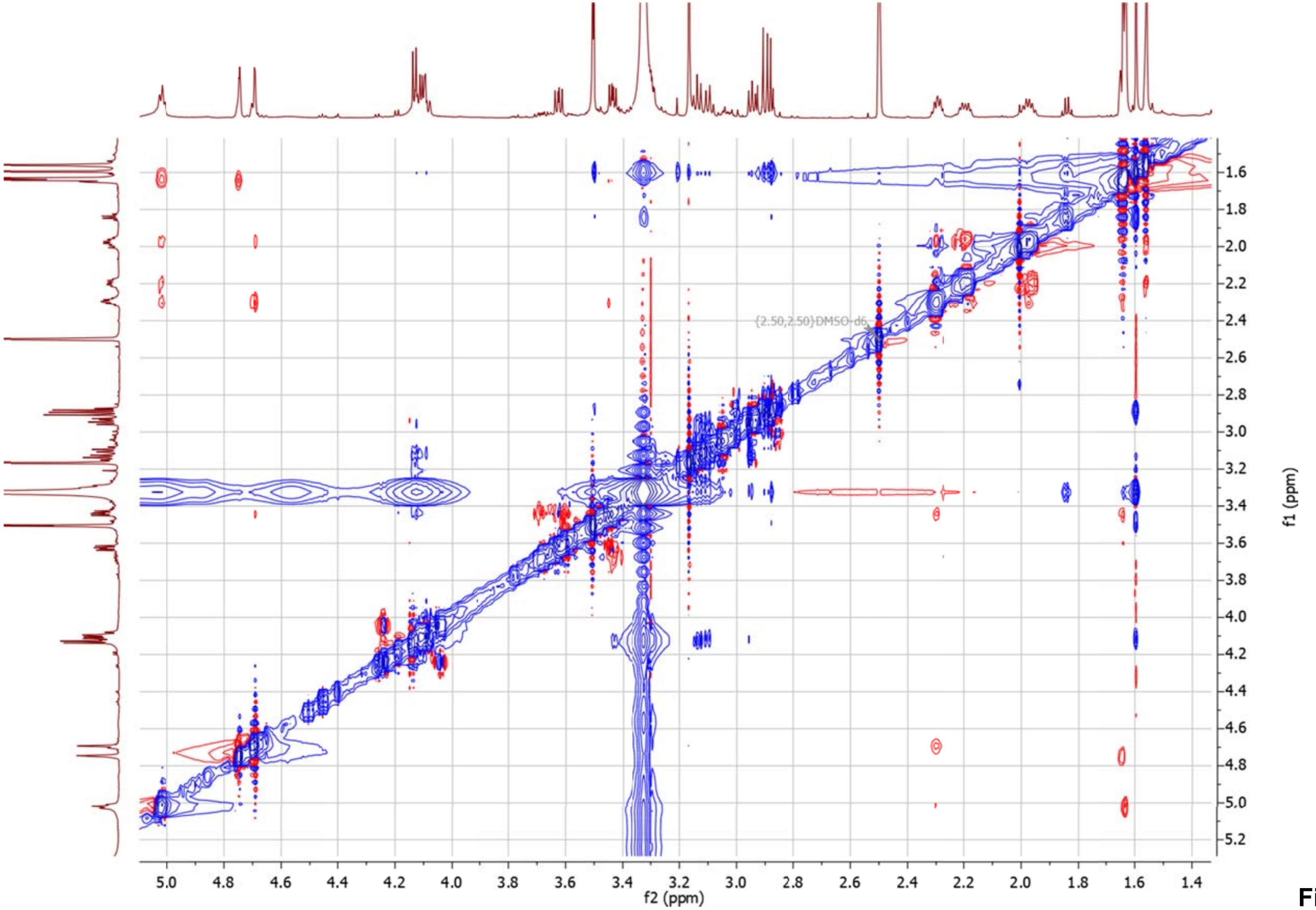

Figure S2.25 1H -1H NOESY spectrum of **3** in DMSO-d6.

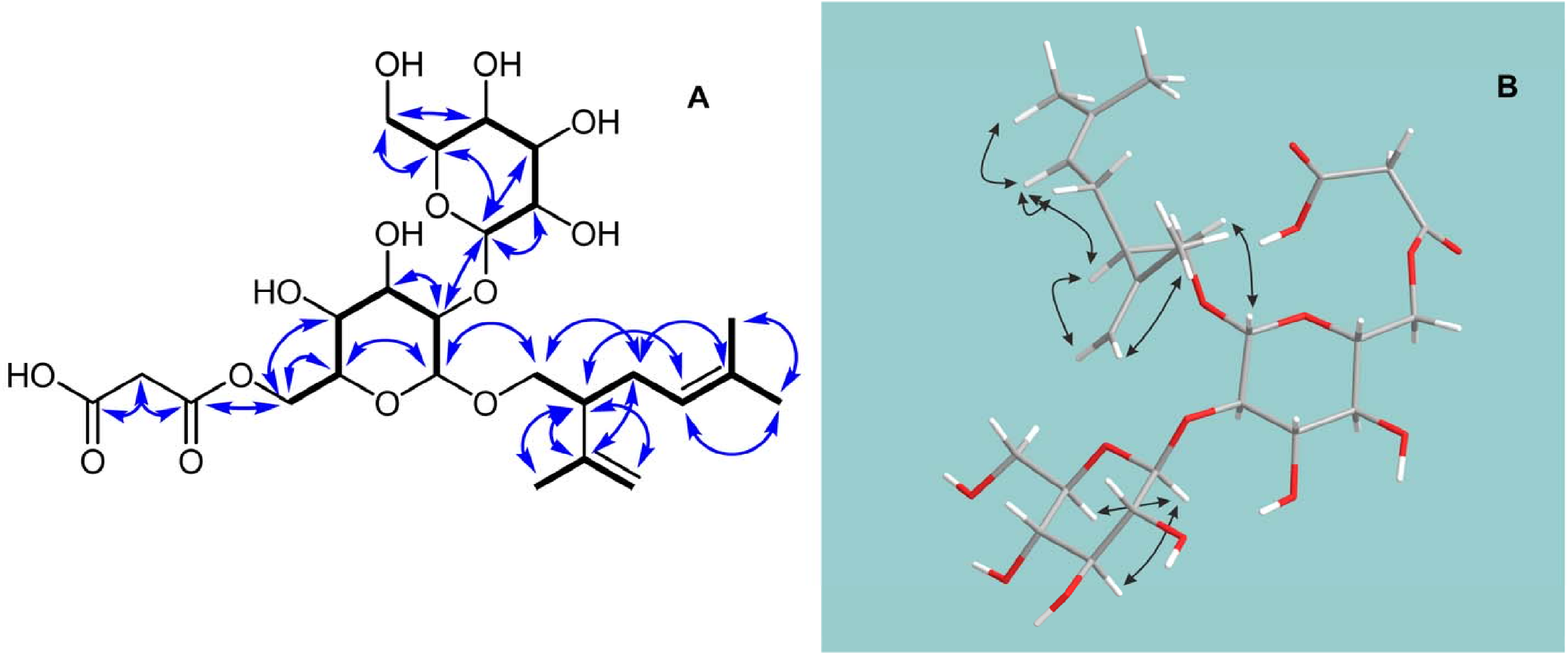

Figure S2.26 COSY (bold lines) and key HMBC (arrows) correlations (A), and NOESY correlations (B) of **4** in DMSO-d_6.

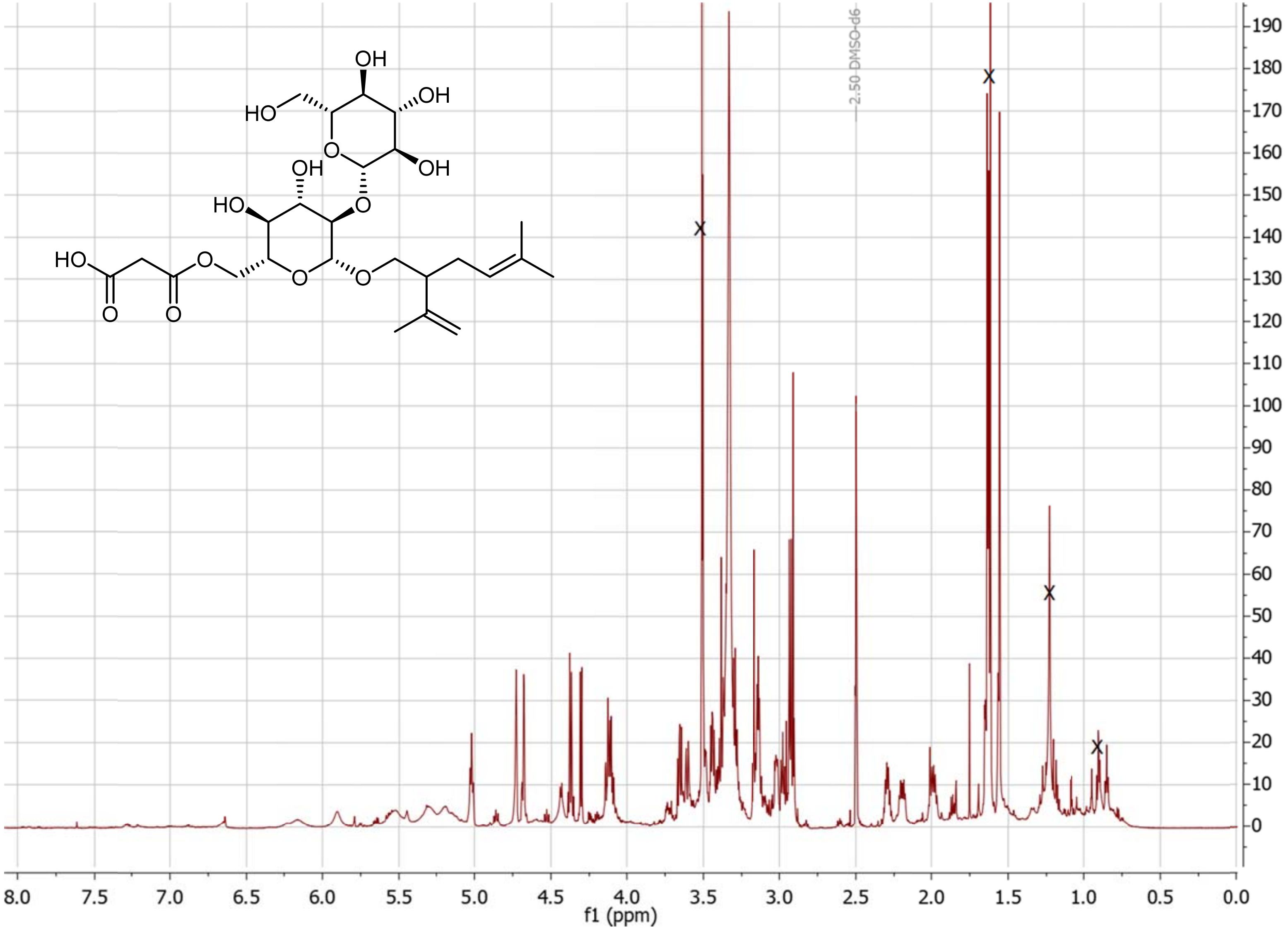

Figure S2.27 ^{1}H NMR spectrum of **4** in DMSO-d_6. The signals representing residual sample impurities are crossed out.

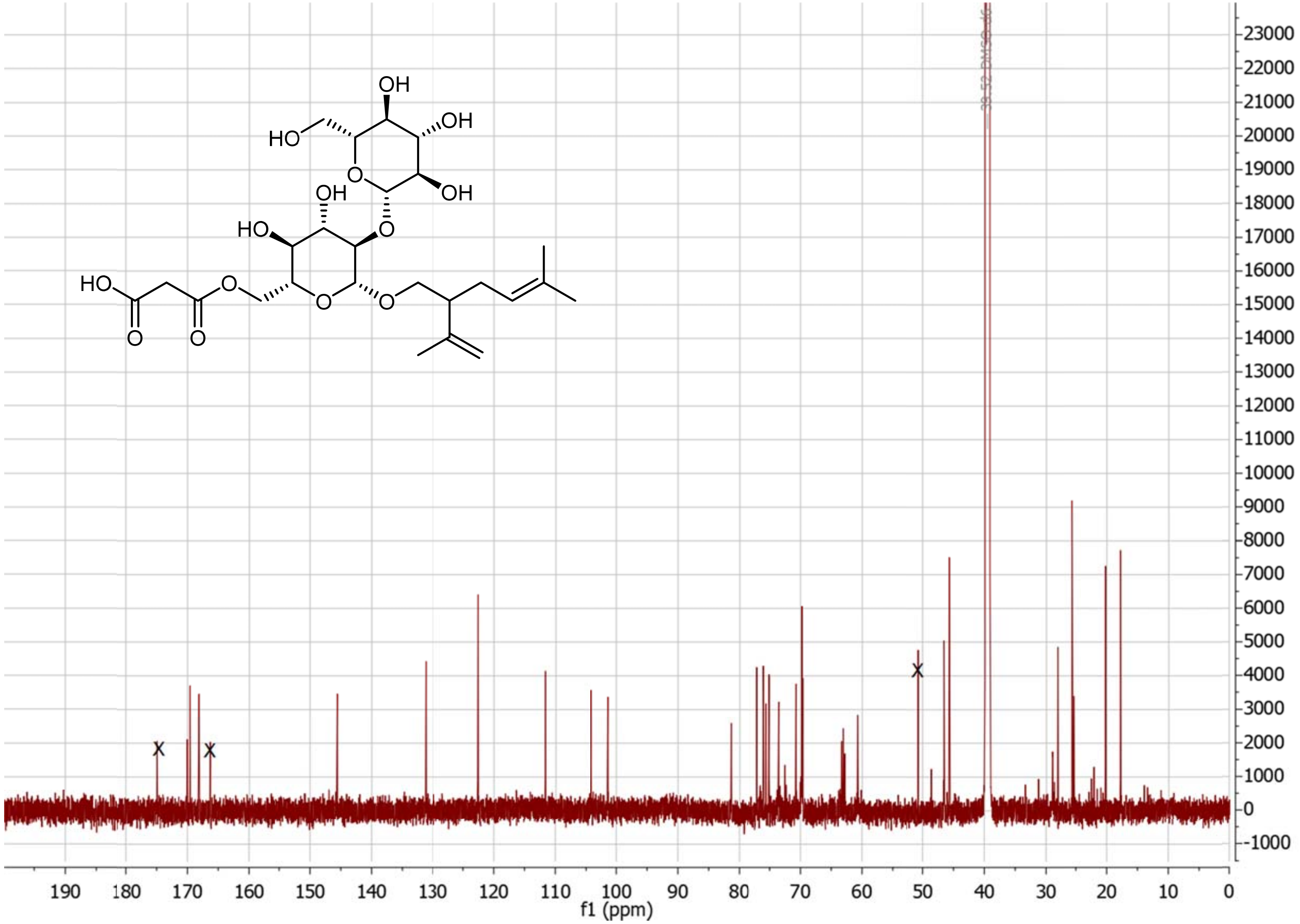

Figure S2.28 ^{13}C NMR spectrum of **4** in DMSO-d_6. The signals representing residual sample impurities are crossed out.

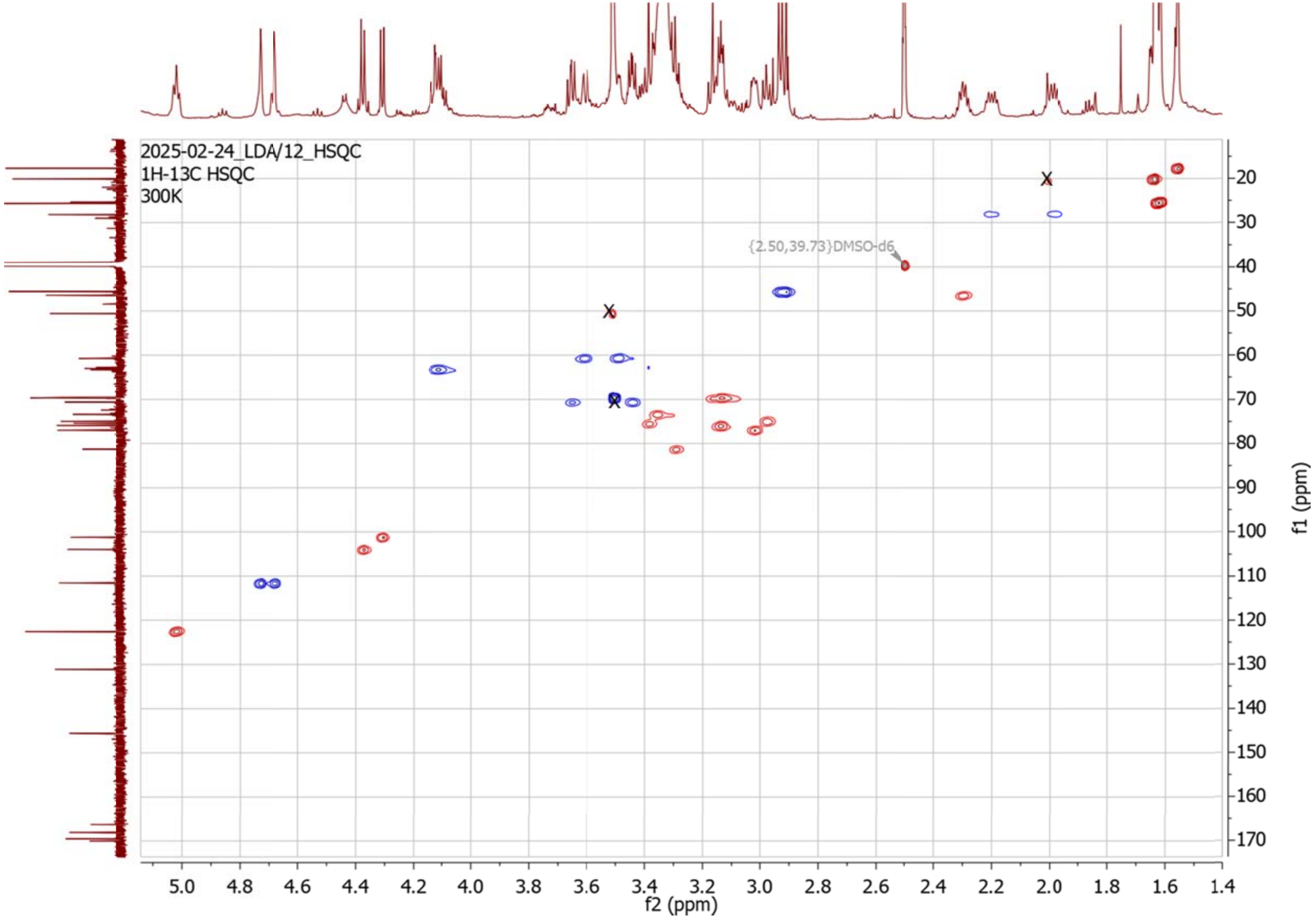

Figure S2.29 ^{1}H -^{13}C HSQC spectrum of **4** in DMSO-d_6. The signals representing residual sample impurities are crossed out.

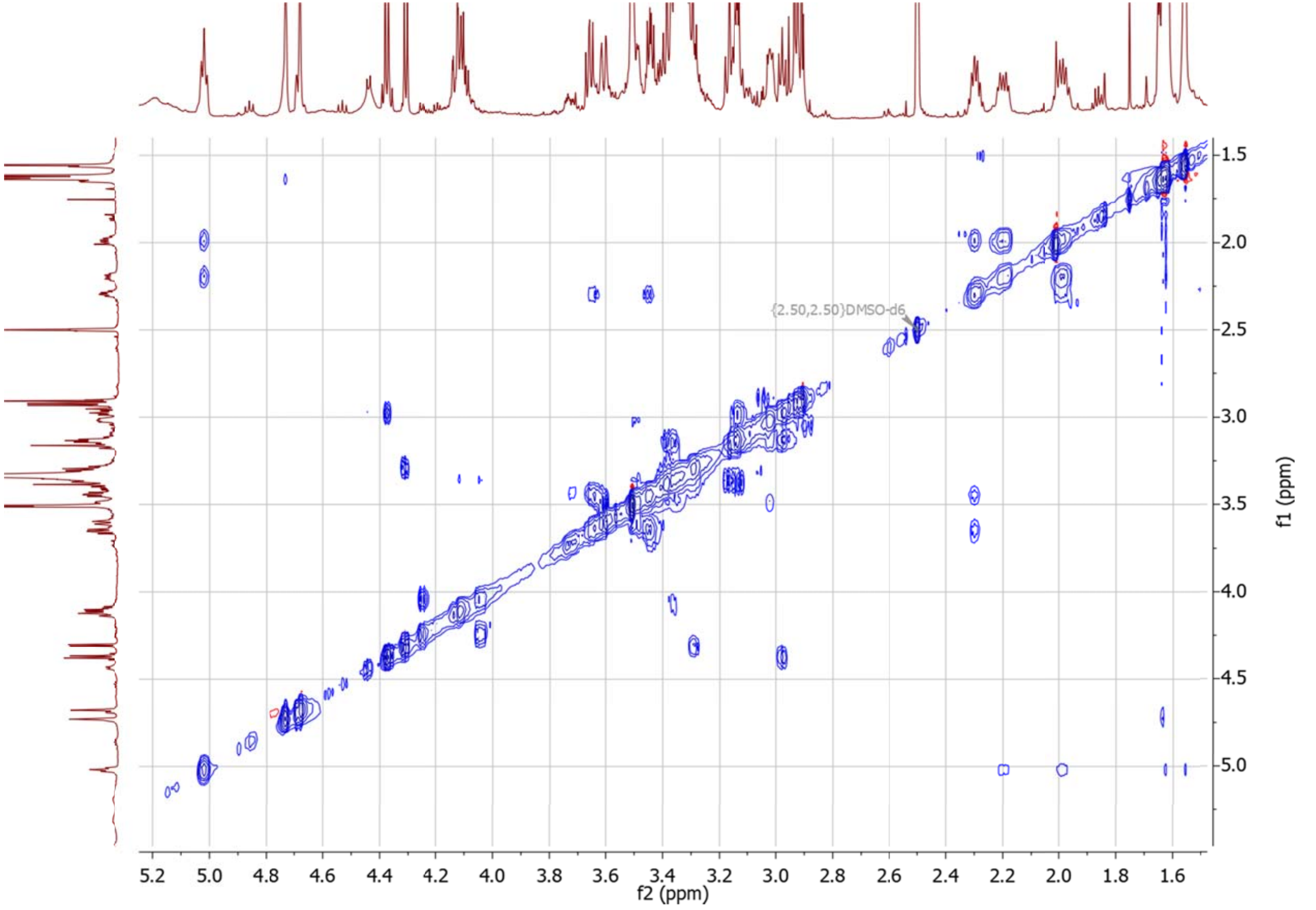

Figure S2.30 ^{1}H -^{1}H CLIP-COSY spectrum of **4** in DMSO-d_6.

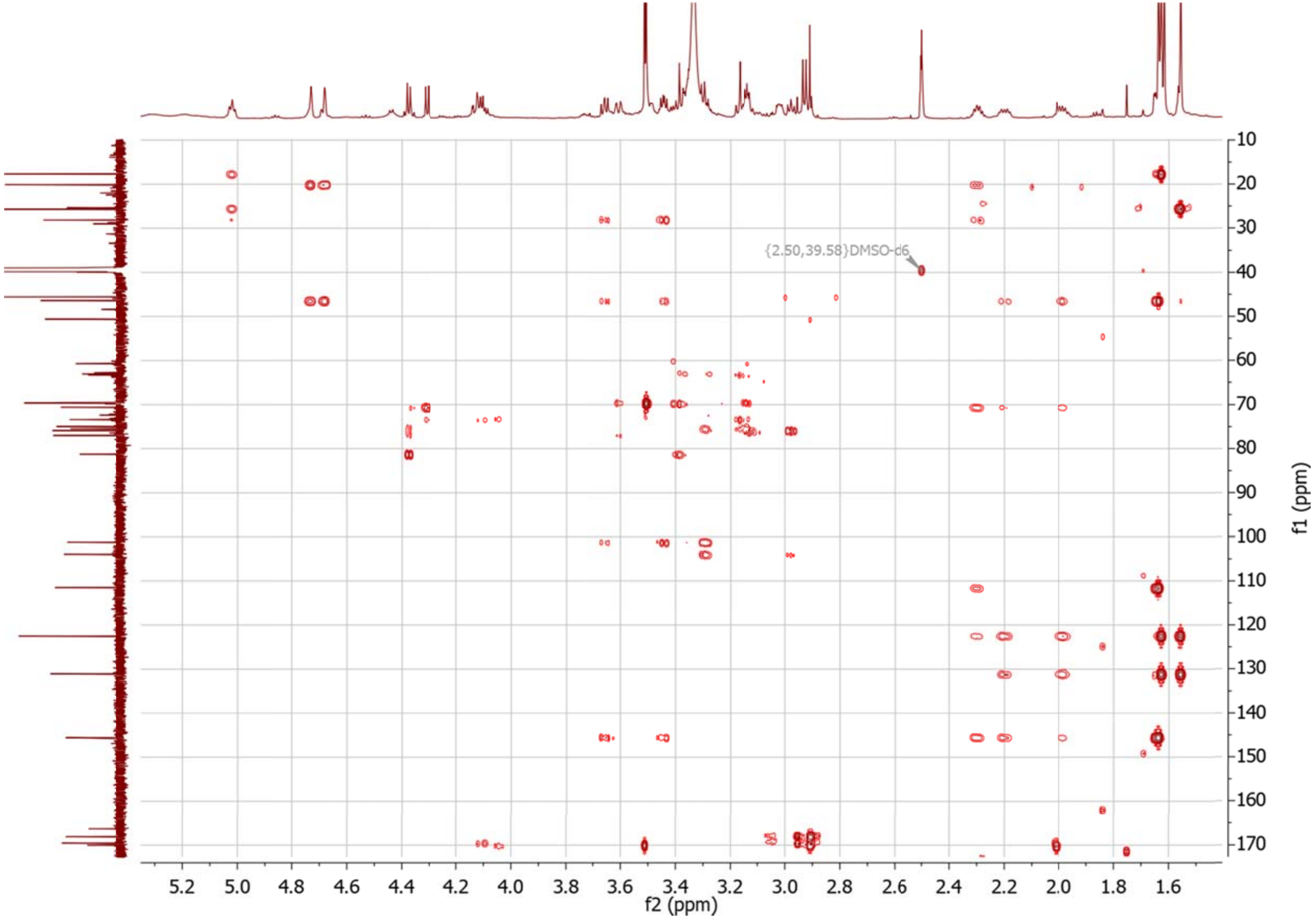

Figure S2.31 ^{1}H -^{13}C HMBC spectrum of **4** in DMSO-d_6.

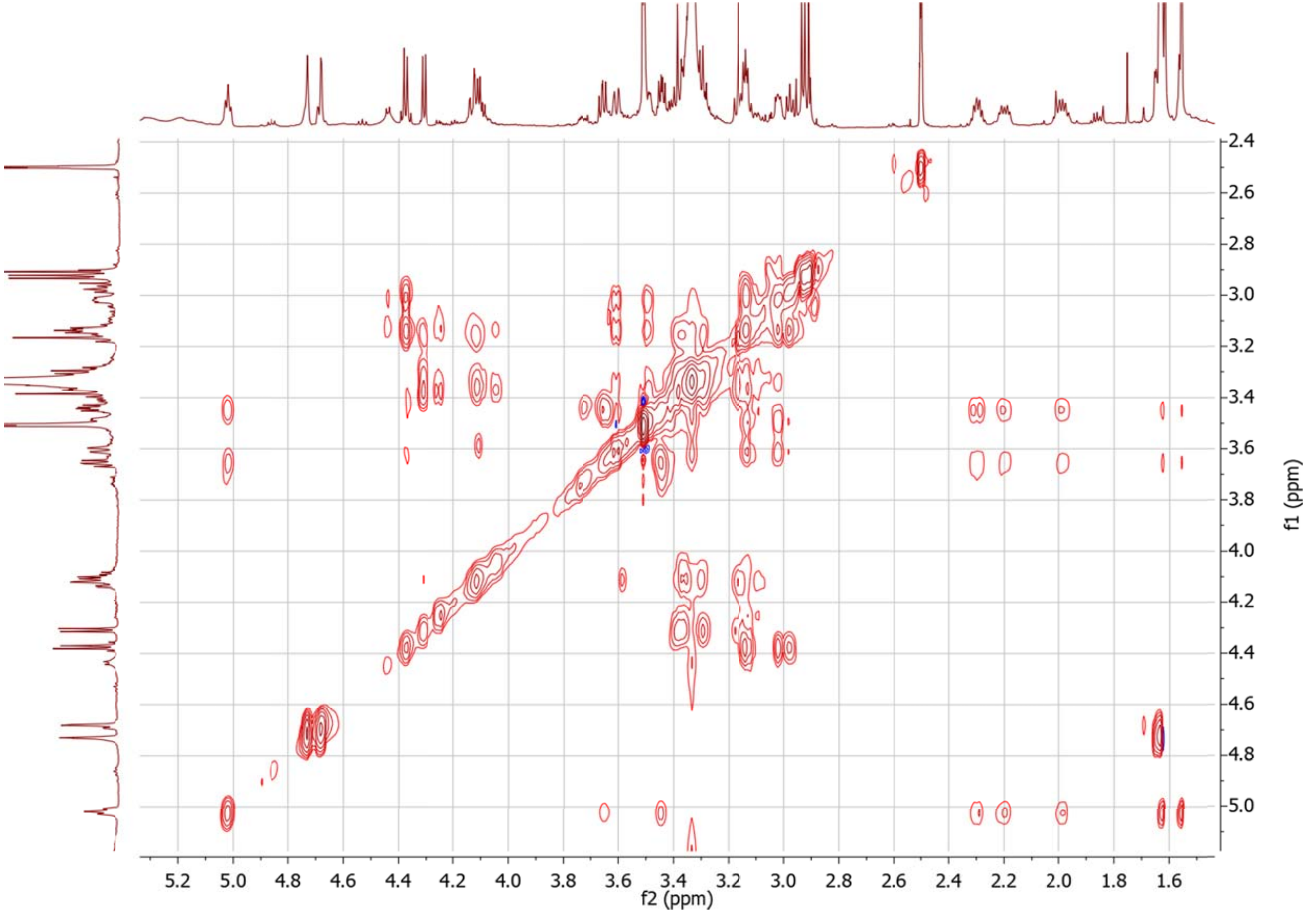

Figure S2.32 ^{1}H -^{1}H TOCSY spectrum of **4** in DMSO-d_6.

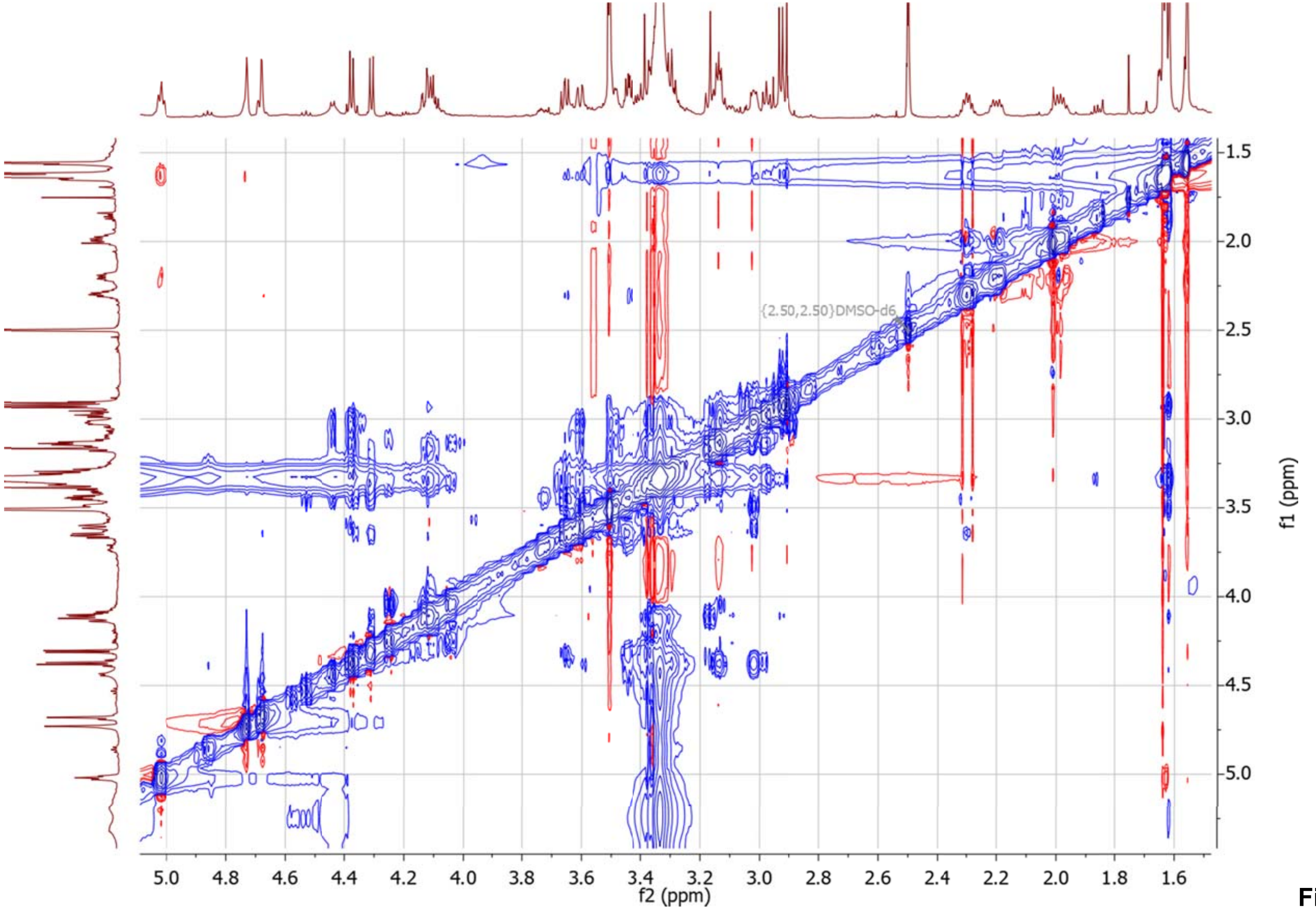

Figure S2.33 ^{1}H -^{1}H NOESY spectrum of **4** in DMSO-d_6.

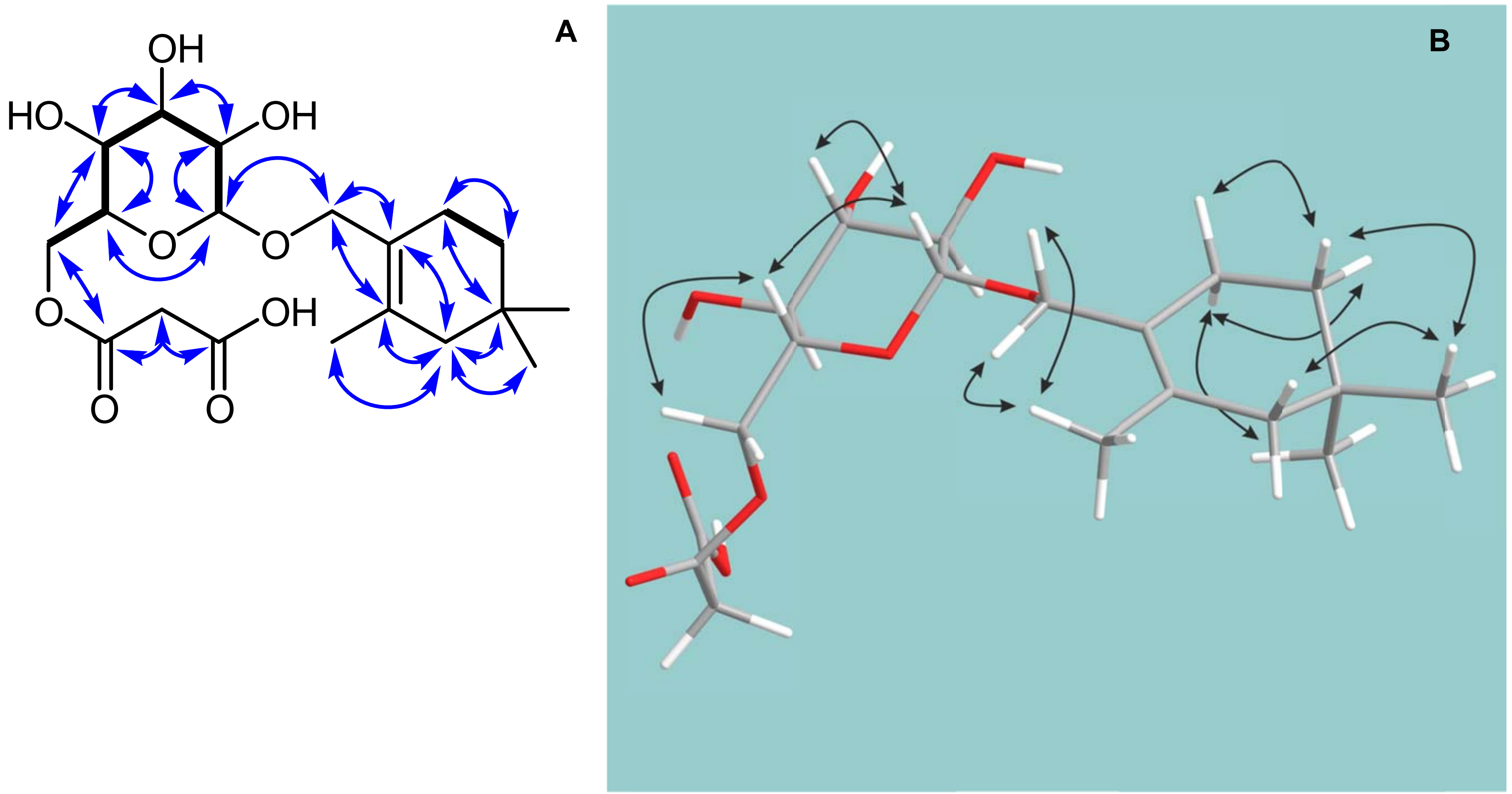

Figure S2.34 COSY (bold lines) and key HMBC (arrows) correlations (A), and NOESY correlations (B) of **5** in DMSO-d_6.

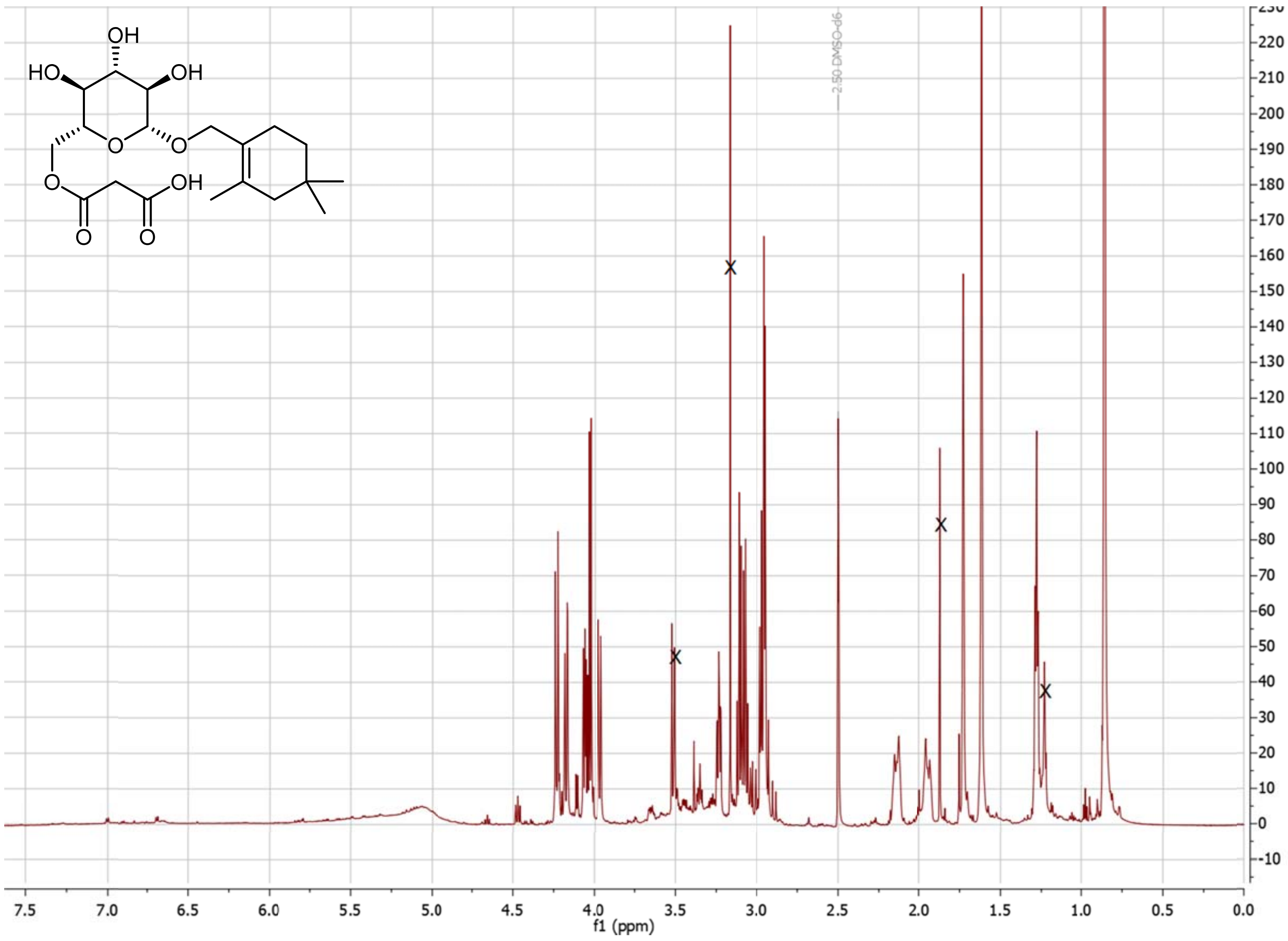

Figure S2.35 ^{1}H NMR spectrum of **5** in DMSO-d_6. The signals representing residual sample impurities are crossed out.

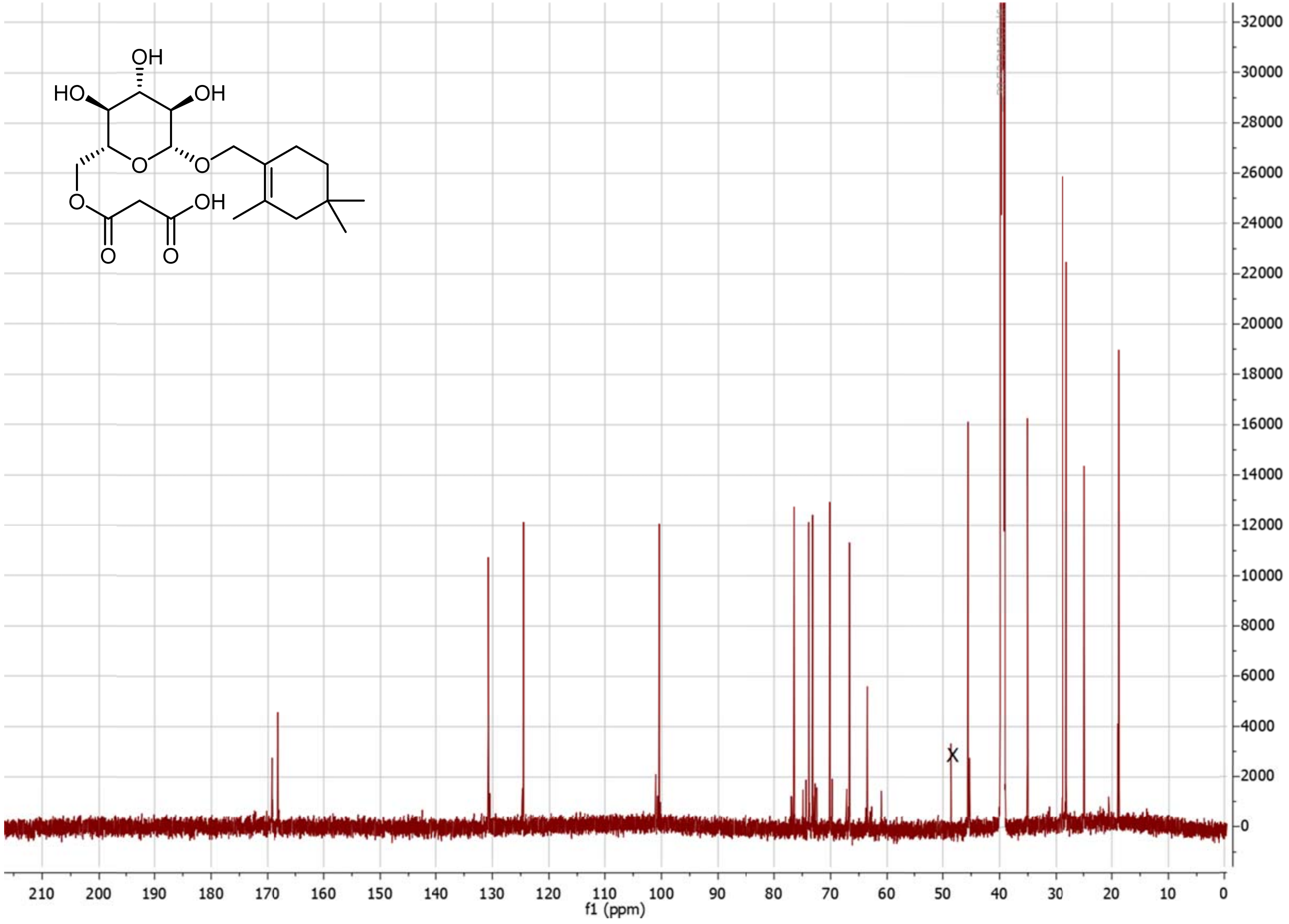

Figure S2.36 ^{13}C NMR spectrum of **5** in DMSO-d_6. The signals representing residual sample impurities are crossed out.

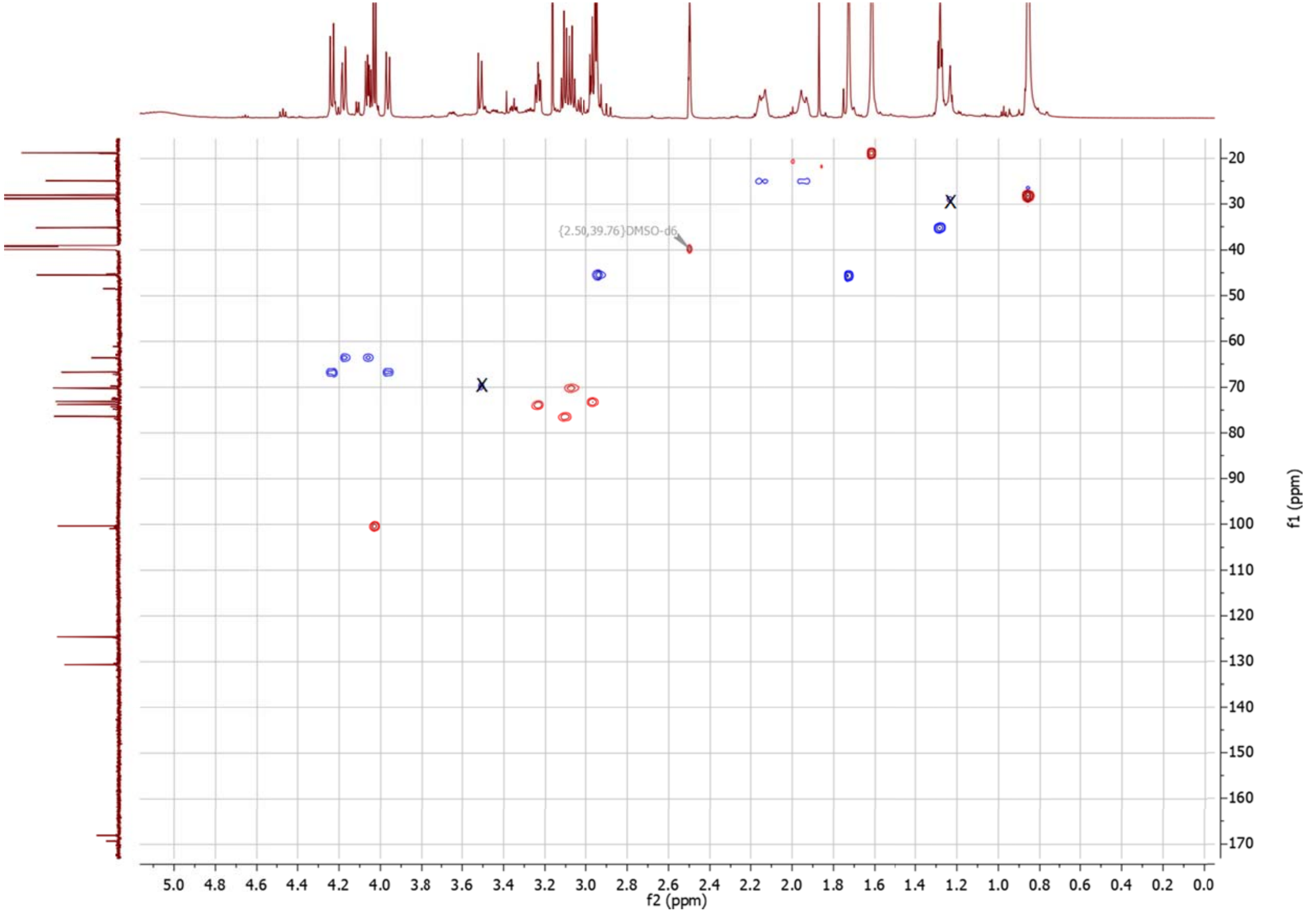

Figure S2.37 ^{1}H -^{13}C HSQC spectrum of **5** in DMSO-d_6. The signals representing residual sample impurities are crossed out.

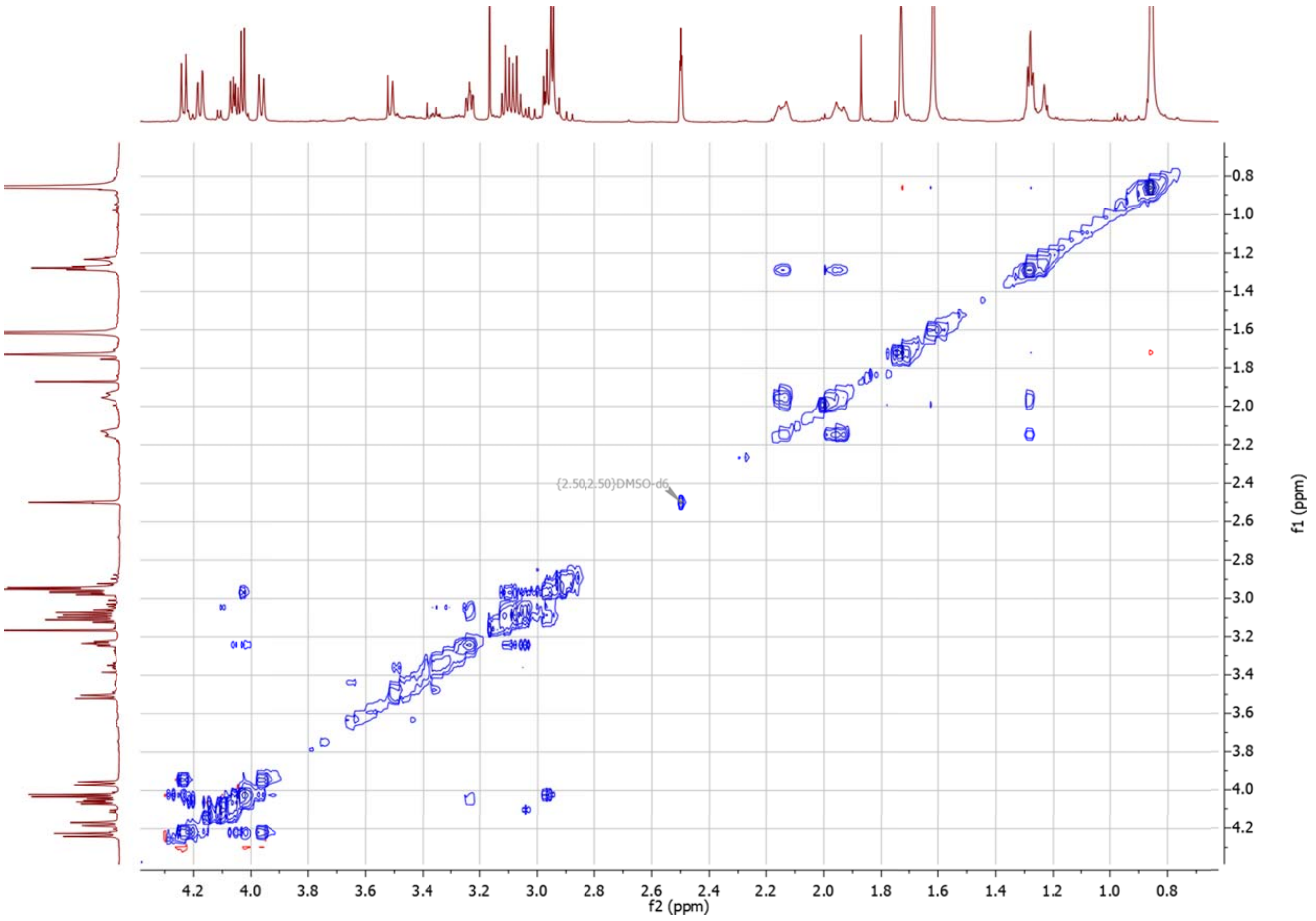

Figure S2.38 ^{1}H -^{1}H CLIP-COSY spectrum of **5** in DMSO-d_6.

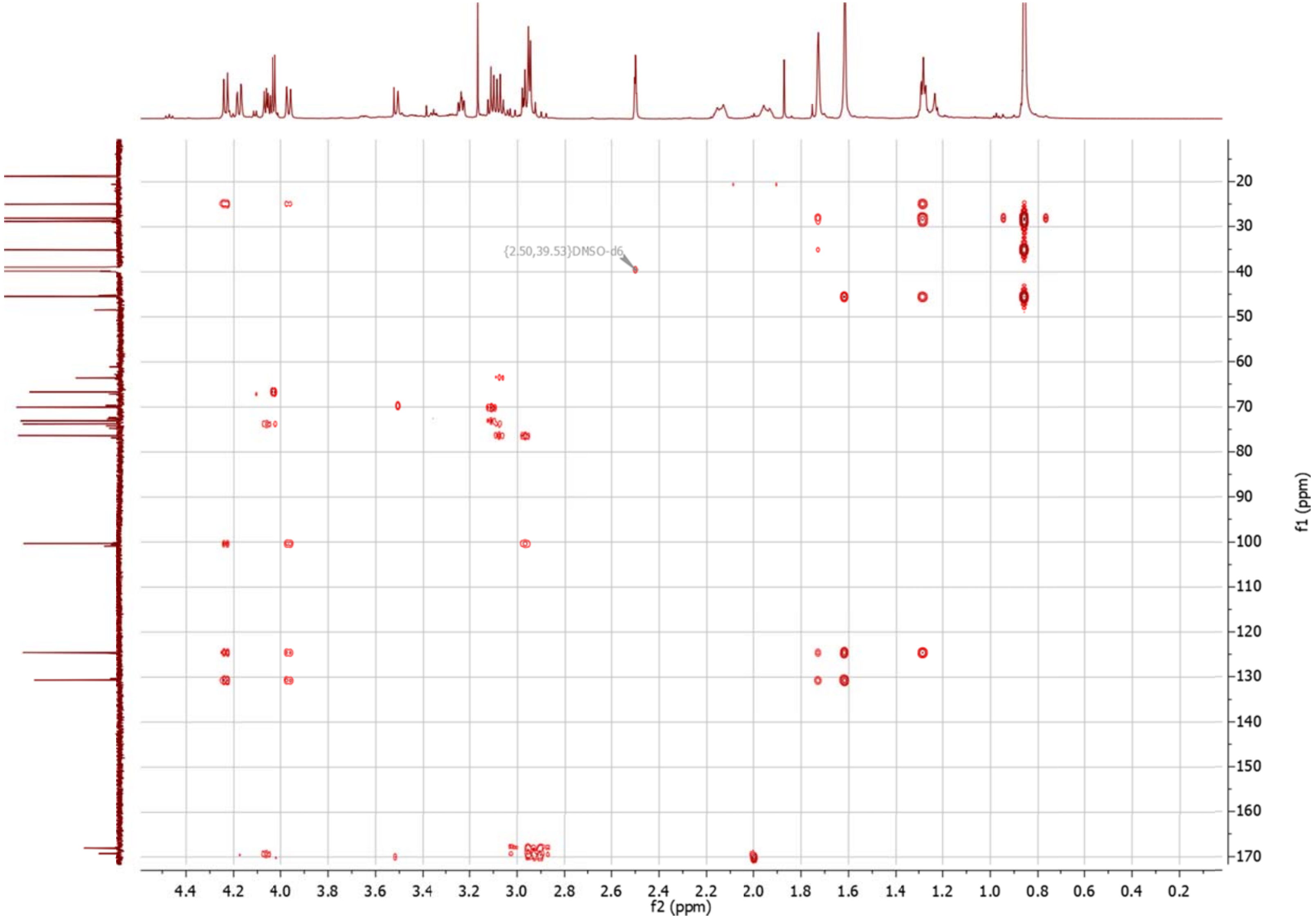

Figure S2.39 ^{1}H -^{13}C HMBC spectrum of **5** in DMSO-d_6.

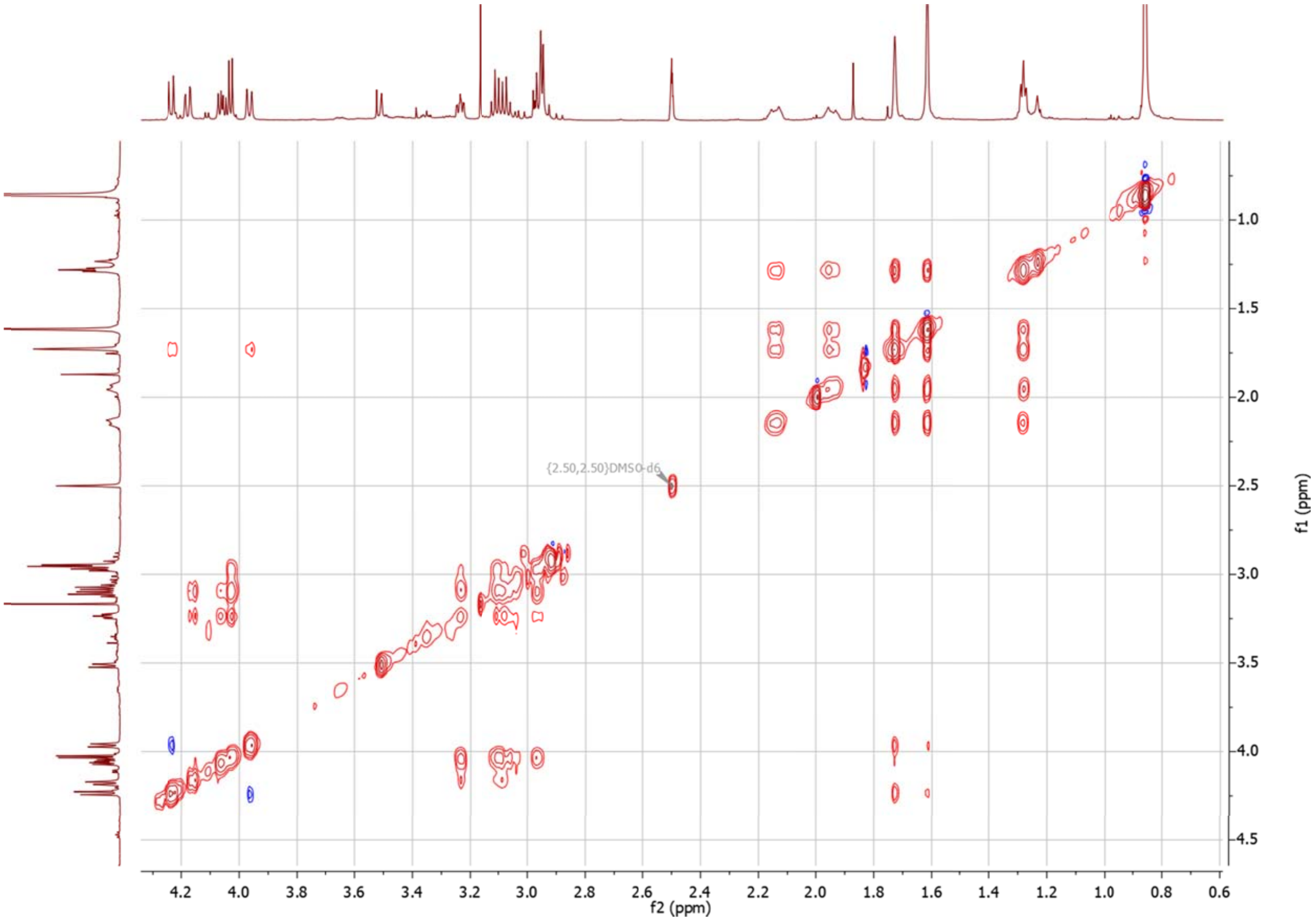

Figure S2.40 ^{1}H -^{1}H TOCSY spectrum of **5** in DMSO-d_6.

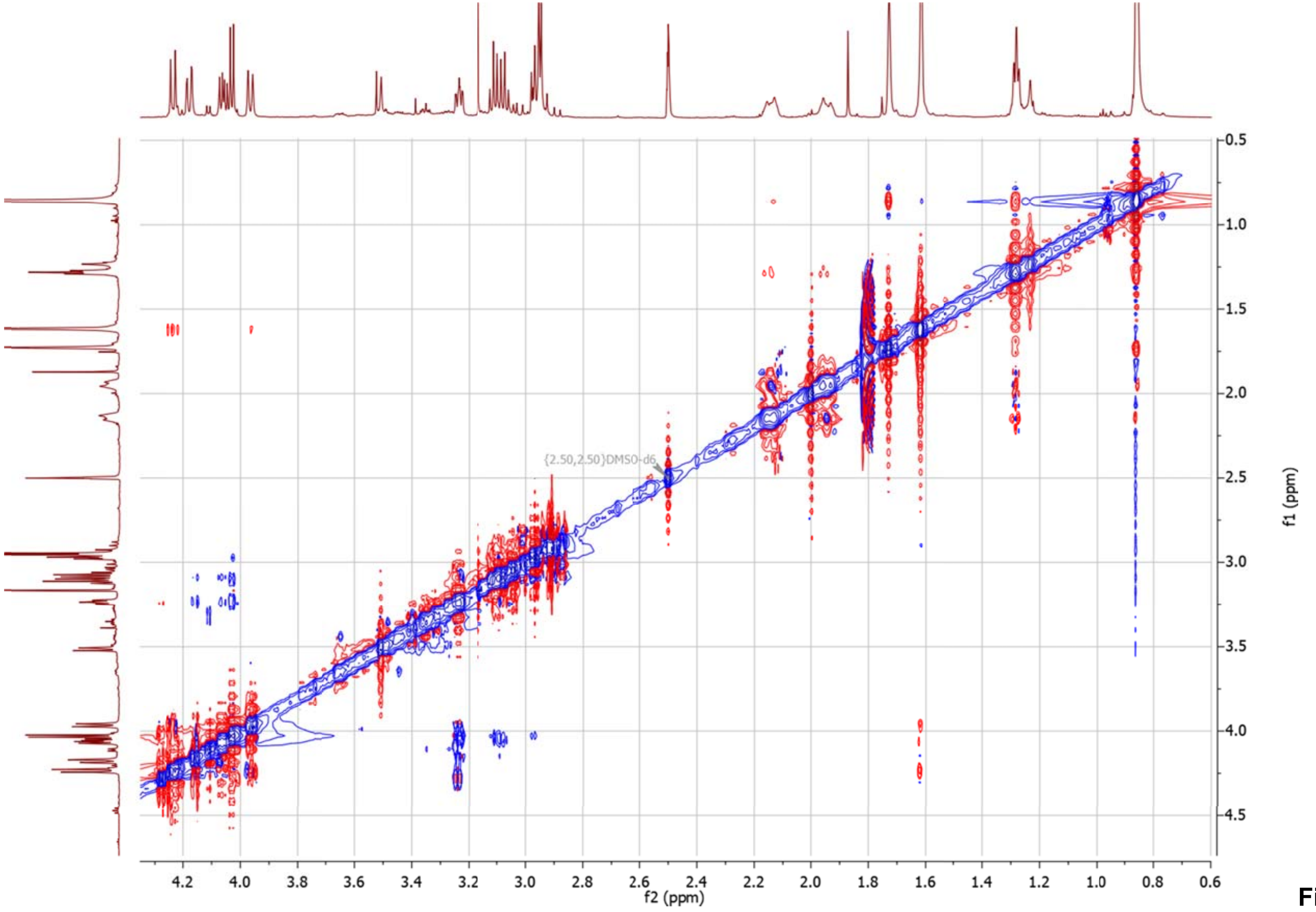

Figure S2.41 ^1H -^1H NOESY spectrum of **5** in DMSO-d_6.

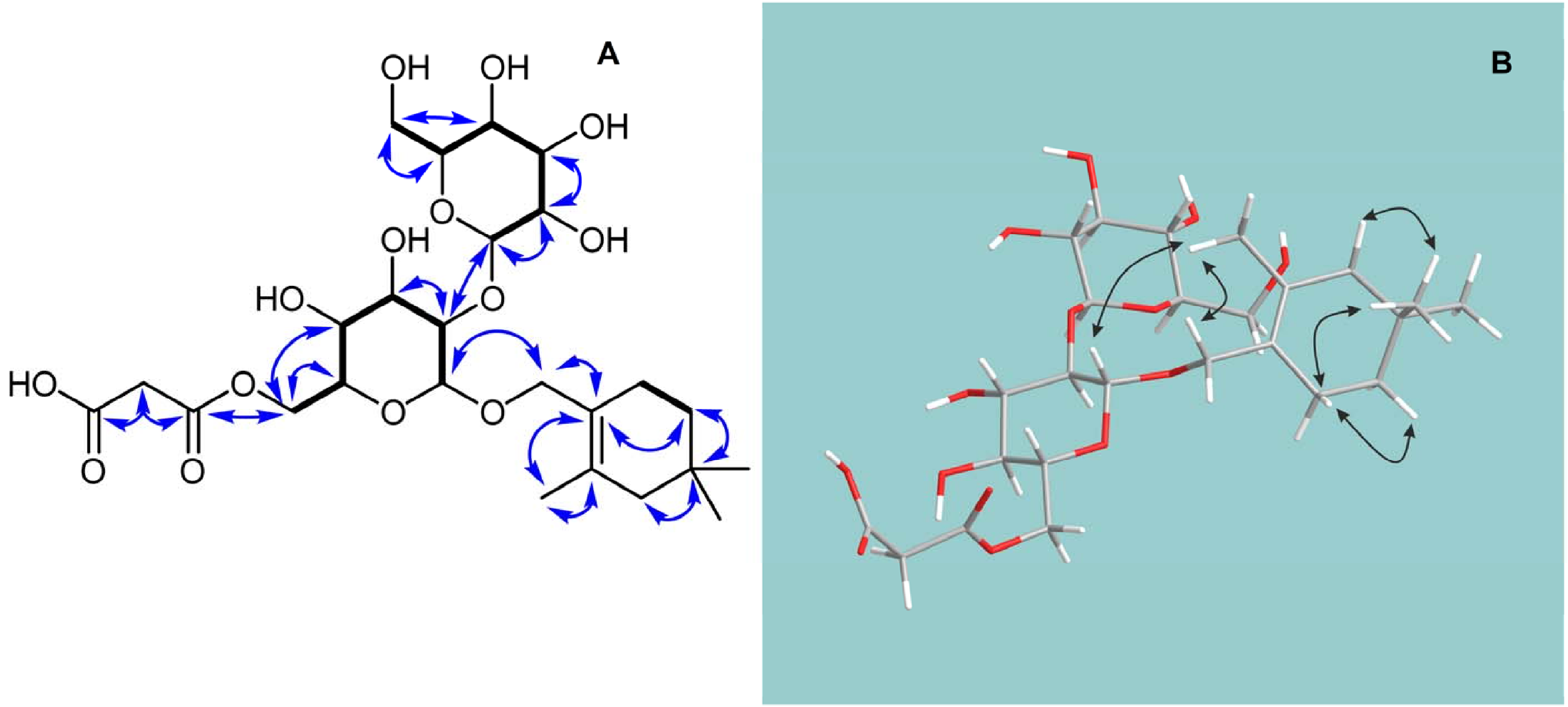

Figure S2.42 COSY (bold lines) and key HMBC (arrows) correlations (A), and NOESY correlations (B) of **6** in DMSO-d_6.

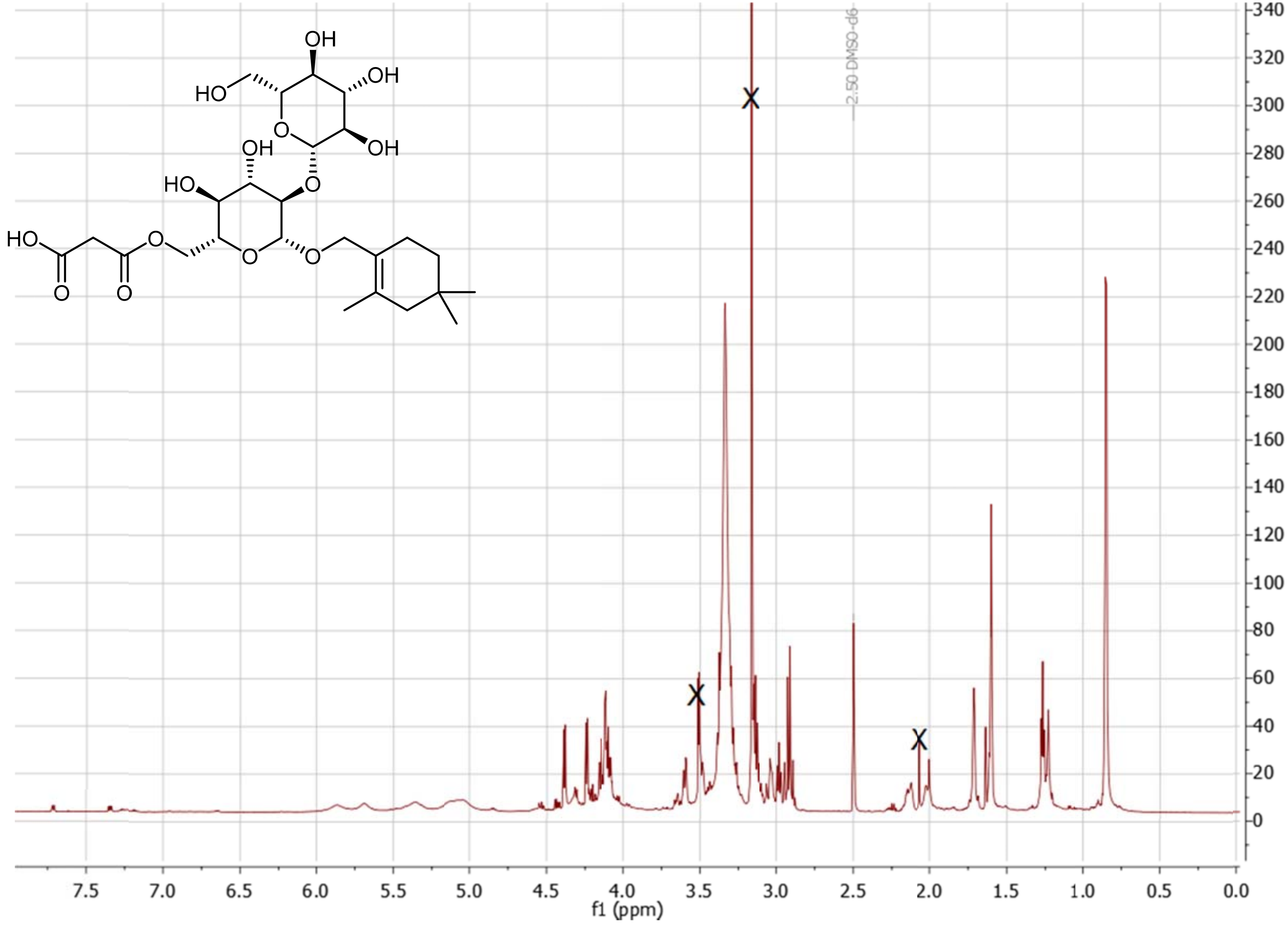

Figure S2.43 ^{1}H NMR spectrum of **6** in DMSO-d_6. The signals representing residual sample impurities are crossed out.

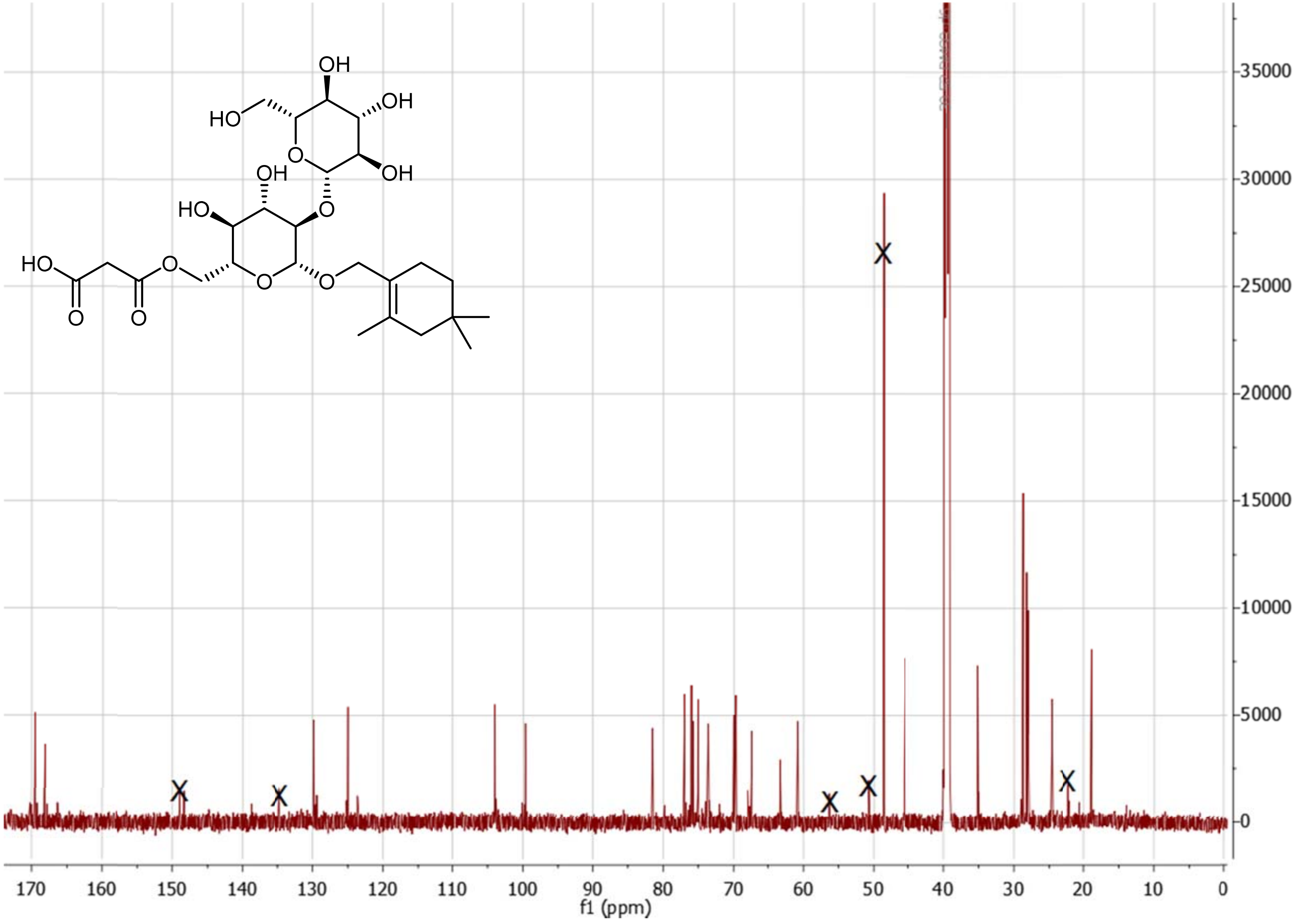

Figure S2.44 ^{13}C NMR spectrum of **6** in DMSO-d_6. The signals representing residual sample impurities are crossed out.

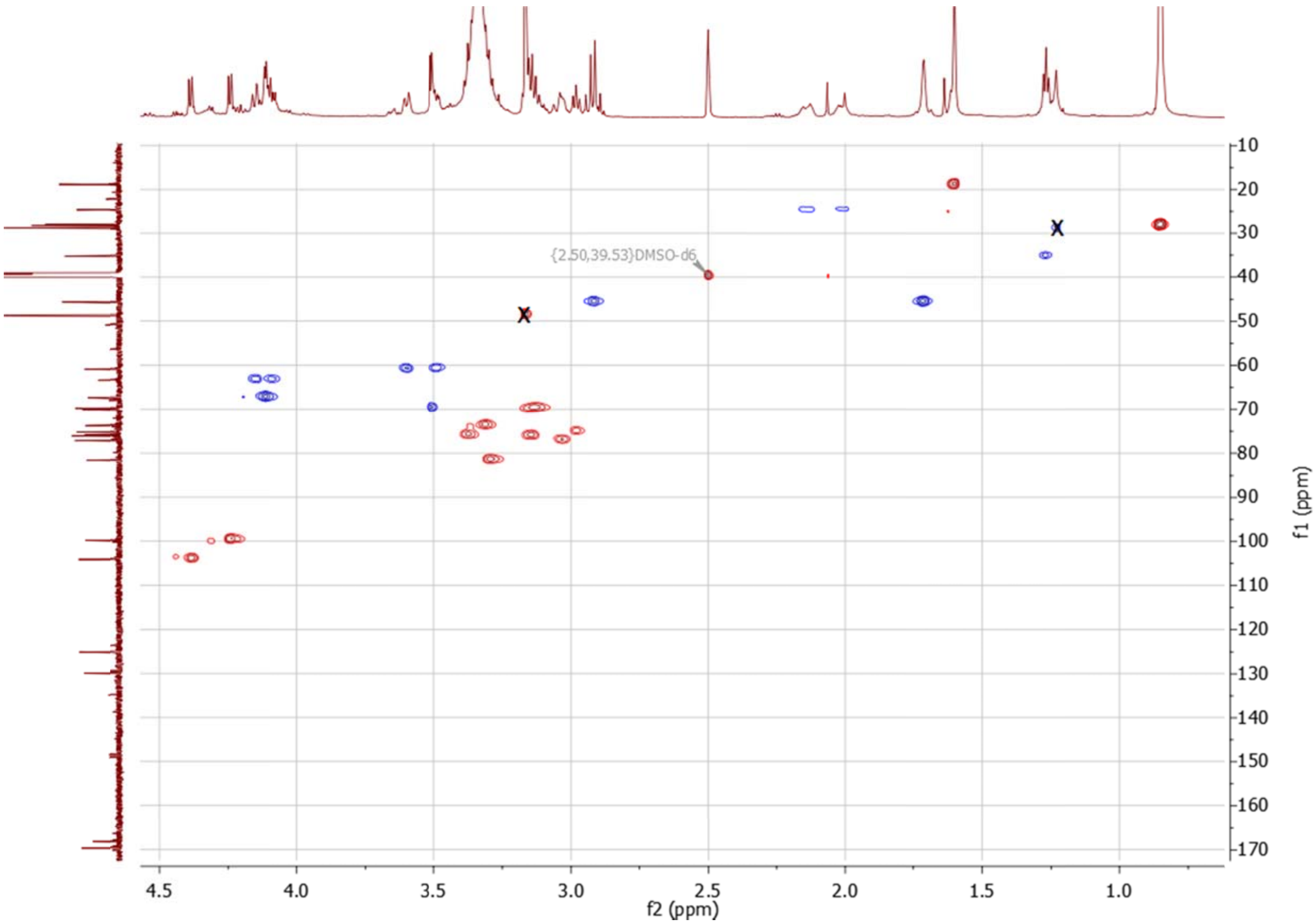

Figure S2.45 ^{1}H -^{13}C HSQC spectrum of **6** in DMSO-d_6. The signals representing residual sample impurities are crossed out.

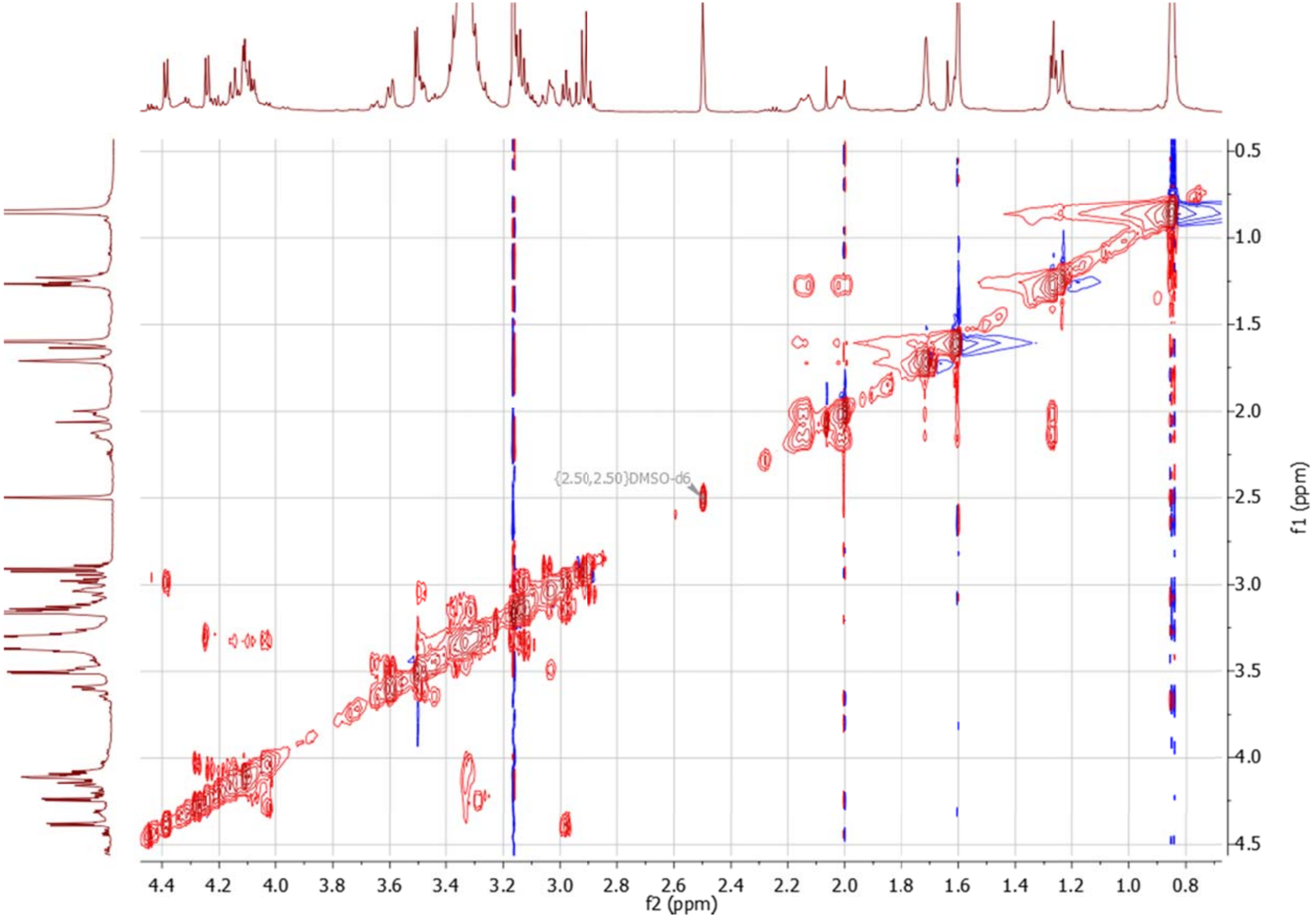

Figure S2.46 ^{1}H -^{1}H CLIP-COSY spectrum of **6** in DMSO-d_6.

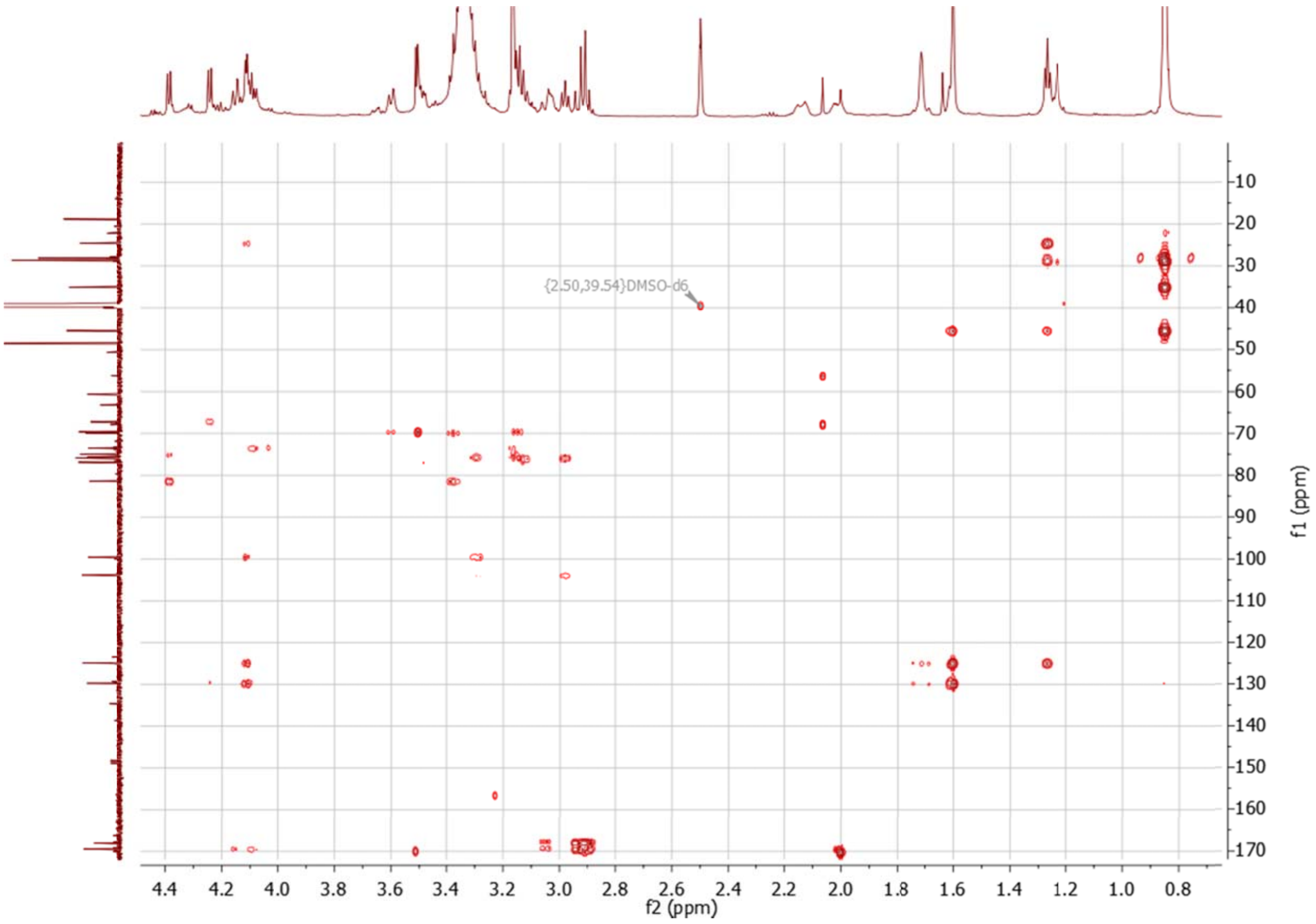

Figure S2.47 ^{1}H -^{13}C HMBC spectrum of **6** in DMSO-d_6.

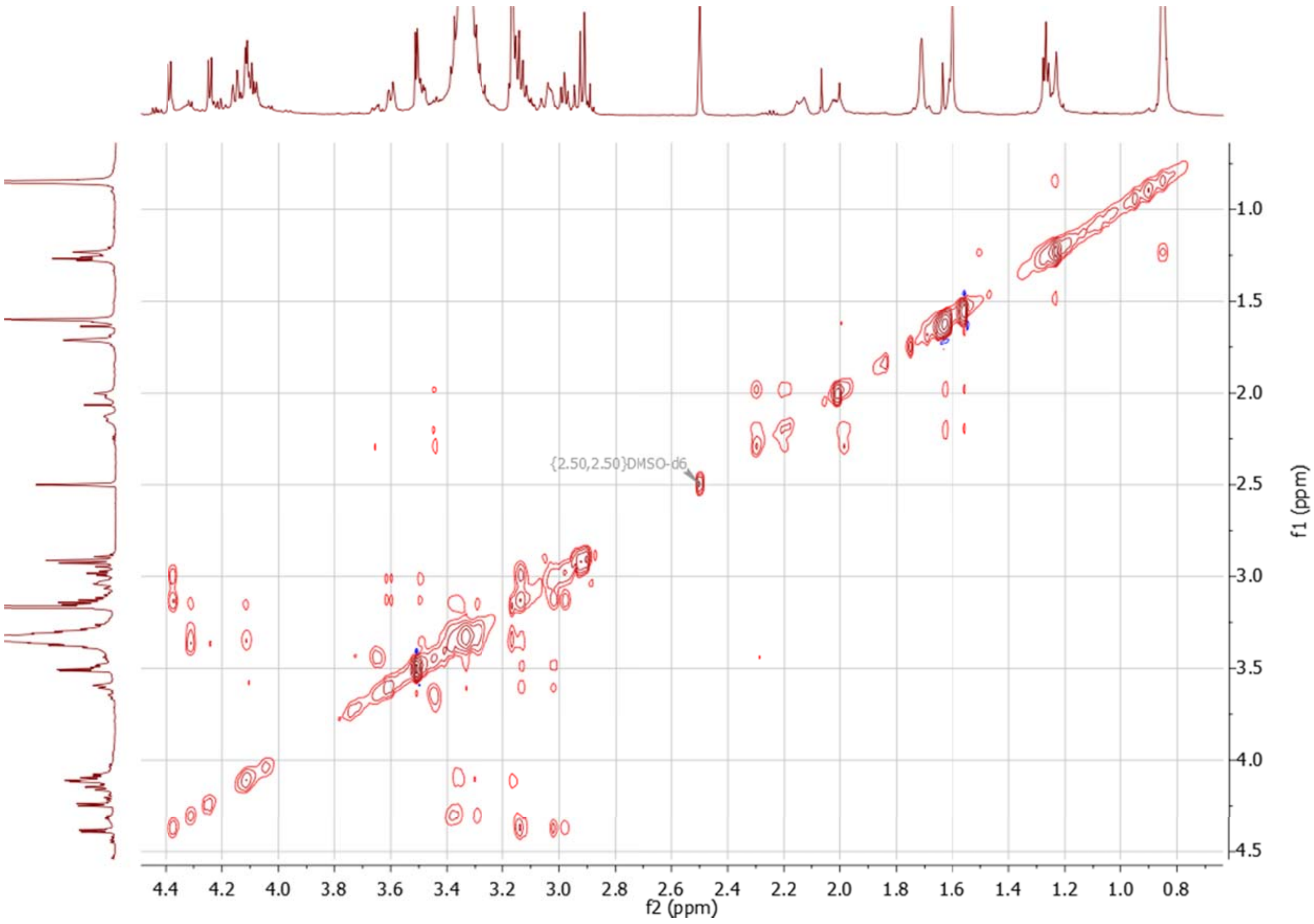

Figure S2.48 ^{1}H -^{1}H TOCSY spectrum of **6** in DMSO-d_6.

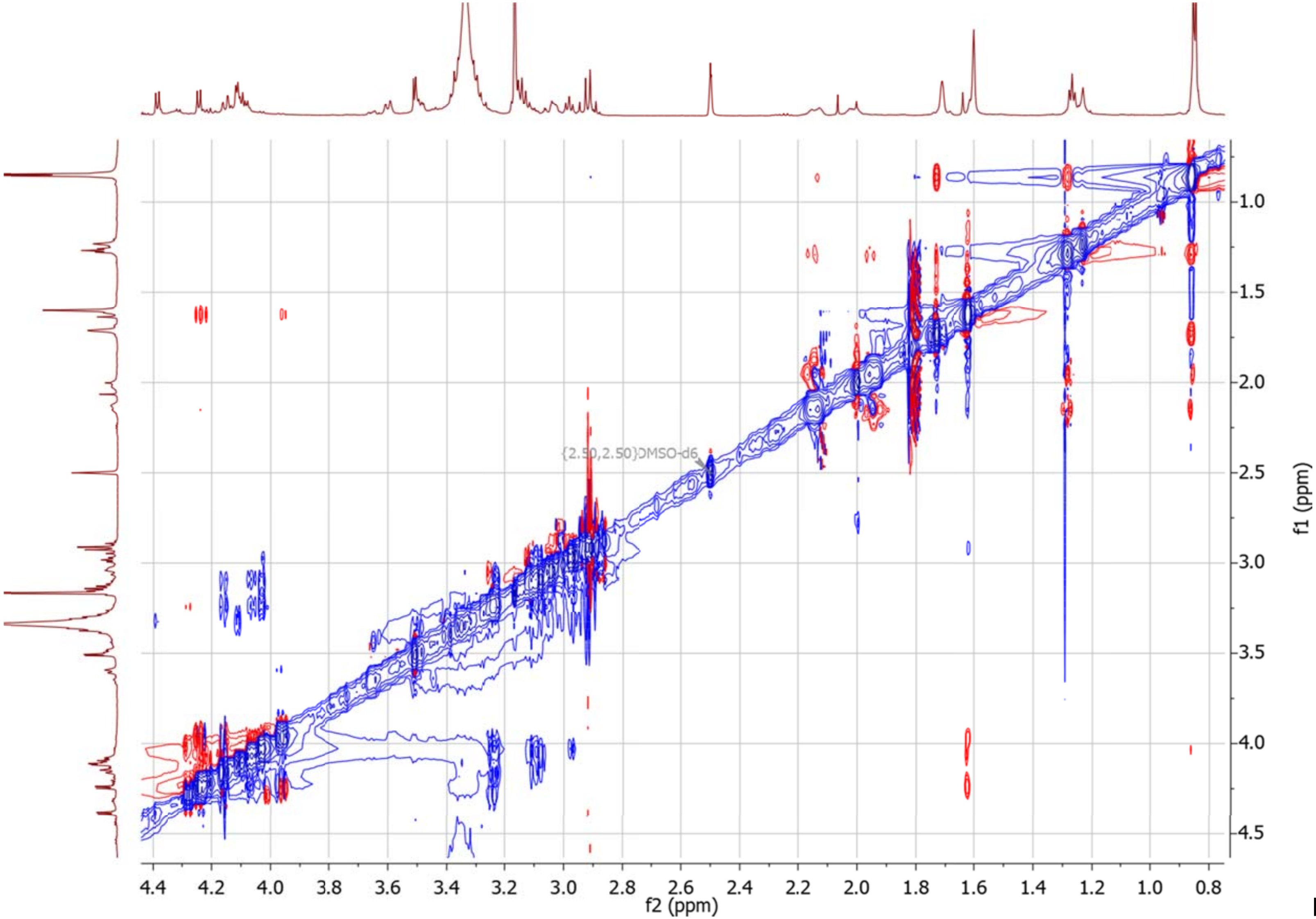

Figure S2.49 ^{1}H -^{1}H NOESY spectrum of **6** in DMSO-d_6.

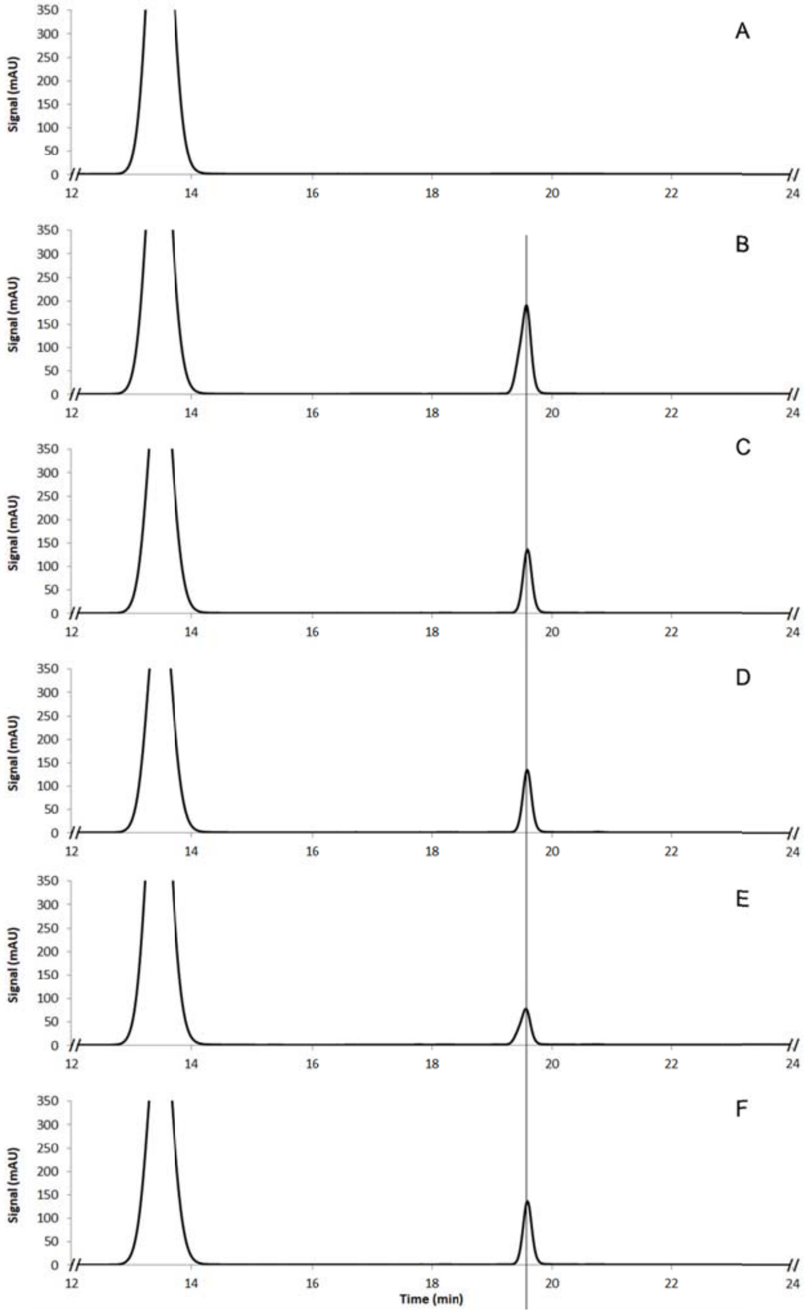

Figure S2.50 Exemplary HPLC separation of the reaction mixture after MPP-derivatization. The following samples are represented: a) Control sample without the substrate; b) Reference sample of D-glucose; c) Sample containing the hydrolysis product of compound **5**; d) Sample **c** mixed with sample **b** in a 1:1 ratio; e) Sample containing the hydrolysis product of compound **6**; f) Sample **e** mixed with sample **b** in a 1:1 ratio.

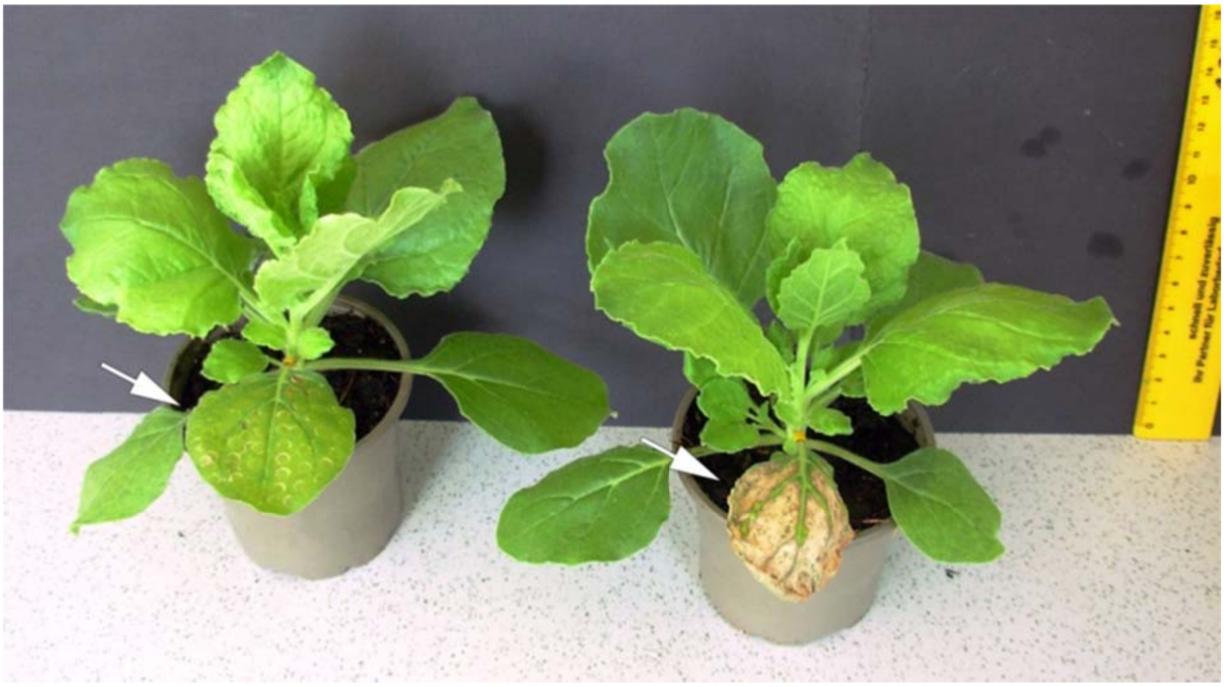

Figure S2.51 *N. benthamiana* plants expressing StCLDS alone and in combination with tHMGR. The infiltrated leaves are indicated by the arrows.
